# Targeting quiescent leukemic stem cells using second generation autophagy inhibitors

**DOI:** 10.1038/s41375-018-0252-4

**Published:** 2018-09-05

**Authors:** Pablo Baquero, Amy Dawson, Arunima Mukhopadhyay, Elodie M. Kuntz, Rebecca Mitchell, Orianne Olivares, Angela Ianniciello, Mary T. Scott, Karen Dunn, Michael C. Nicastri, Jeffrey D. Winkler, Alison M. Michie, Kevin M. Ryan, Christina Halsey, Eyal Gottlieb, Erin P. Keaney, Leon O. Murphy, Ravi K. Amaravadi, Tessa L. Holyoake, G. Vignir Helgason

**Affiliations:** 1 Wolfson Wohl Cancer Research Centre, Institute of Cancer Sciences, University of Glasgow, Glasgow, G61 1QH UK; 2 Paul O’Gorman Leukemia Research Centre, Institute of Cancer Sciences, University of Glasgow, Glasgow, G12 0ZD UK; 3 Cancer Research UK, Beatson Institute, Garscube Estate, Glasgow, G61 1BD UK; 4 Department of Medicine and Abramson Cancer Center, Perelman School of Medicine, University of Pennsylvania, Philadelphia, PA USA; 5 Novartis Institutes for BioMedical Research, 250 Massachusetts Avenue, Cambridge, Massachusetts, 02139 USA

**Keywords:** Chronic myeloid leukaemia, Cancer stem cells

## Abstract

In chronic myeloid leukemia (CML), tyrosine kinase inhibitor (TKI) treatment induces autophagy that promotes survival and TKI-resistance in leukemic stem cells (LSCs). In clinical studies hydroxychloroquine (HCQ), the only clinically approved autophagy inhibitor, does not consistently inhibit autophagy in cancer patients, so more potent autophagy inhibitors are needed. We generated a murine model of CML in which autophagic flux can be measured in bone marrow-located LSCs. In parallel, we use cell division tracing, phenotyping of primary CML cells, and a robust xenotransplantation model of human CML, to investigate the effect of Lys05, a highly potent lysosomotropic agent, and PIK-III, a selective inhibitor of VPS34, on the survival and function of LSCs. We demonstrate that long-term haematopoietic stem cells (LT-HSCs: Lin^−^Sca-1^+^c-kit^+^CD48^−^CD150^+^) isolated from leukemic mice have higher basal autophagy levels compared with non-leukemic LT-HSCs and more mature leukemic cells. Additionally, we present that while HCQ is ineffective, Lys05-mediated autophagy inhibition reduces LSCs quiescence and drives myeloid cell expansion. Furthermore, Lys05 and PIK-III reduced the number of primary CML LSCs and target xenografted LSCs when used in combination with TKI treatment, providing a strong rationale for clinical use of second generation autophagy inhibitors as a novel treatment for CML patients with LSC persistence.

## Introduction

Chronic myeloid leukemia (CML) arises following a reciprocal chromosomal translocation within a haematopoietic stem cell (HSC) leading to expression of the fusion oncoprotein BCR-ABL. Despite the significant increase in life expectancy of CML patients due to the development of BCR-ABL-targeting tyrosine kinase inhibitors (TKIs) [1], a quarter of patients will fail TKI therapy due to BCR-ABL kinase mutations, alternative oncogene activation, or because of progression to accelerated phase or blast crisis [2]. Additionally, leukemic stem cells (LSCs) are insensitive to TKIs [3, 4], giving rise to disease persistence and reducing the possibility of successful treatment-free remission (TFR) to only 10–20% [5]. Although this figure may rise with second generation TKIs, the majority of CML patients will require lifelong TKI therapy. Therefore, a key aim is to identify critical survival mechanisms in LSCs, such that LSC-targeting interventions can be developed, thus increasing the proportion of patients that achieve sustained deep molecular responses and TFR.

Autophagy is an evolutionarily conserved catabolic process used to recycle cytoplasmic material. This process is enabled through the formation of a double membrane vesicle called an autophagosome, which transports cellular material to lysosomes for degradation, and allows cells to maintain cellular homeostasis under basal conditions and ensure survival after exposure to stress factors [6–8].

The evidence that autophagy plays predominantly a cytoprotective role in the context of cancer therapy has paved the way for testing autophagy inhibition as a new therapeutic strategy. The lysosomotropic agent hydroxychloroquine (HCQ), has been shown to inhibit autophagy in preclinical cancer models [9]. We have previously shown that high concentration (≥10 µM) of HCQ sensitizes LSCs to TKI treatment in vitro [10, 11]; however, concern about the potency of HCQ in cancer patients [12–16] has promoted the development of more selective and potent compounds with similar chemical and lysosomotropic properties to HCQ. Furthermore, a suitable model for visualizing and measuring the effect of autophagy inhibitors in vivo has been lacking and, therefore, the biological effects of autophagy inhibition on the maintenance and function of bone marrow (BM)-localized LSCs is currently unknown.

We previously demonstrated that the bivalent aminoquinoline Lys05, a dimeric analogue of chloroquine, is 3 to 10-fold more potent as an autophagy inhibitor than HCQ in cancer cell lines [17]. Another strategy to inhibit autophagy is targeting specific proteins involved in the formation of the autophagosome such as the class III phosphatidylinositol 3-kinase, vacuolar protein sorting 34 (VPS34). VPS34 is required to generate phosphatidylinositol(3)-phosphate for the recruitment of other autophagy-related (ATG) proteins to the nascent autophagosome membrane. Recently, selective inhibitors of VPS34 kinase function have been described [18–20] including PIK-III, which blocks de novo lipidation of the key autophagosome component microtubule-associated protein 1 light chain 3 (LC3) and prevents cargo degradation [20].

In this study, we generated a transgenic murine model by crossing a tetracycline-regulated CML model [21], with a mouse bearing the autophagy marker LC3 fused to GFP [22], which allowed accurate assessment of autophagic vesicle accumulation in LSCs in vivo. Using this model, in parallel with primary stem-cell enriched CML samples and a patient-derived xenograft (PDX) model, we demonstrate that Lys05 and PIK-III-mediated autophagy inhibition reduces LSC quiescence and drives myeloid progenitor cell expansion. Of clinical significance, we show that Lys05 or PIK-III, when combined with TKIs, selectively target LSCs providing a novel rationale to eradicate cancer stem cells in CML patients with persistent disease.

## Materials and methods

### In vivo studies

Inducible *Scl-tTa–BCR-ABL* mice (C57Bl6 background), *Atg7*^*flox/flox*^*:Mx-Cre* (C57Bl6/129Sv1) and *GFP-LC3* (C57Bl6) mice were generated as previously described [21–23]. For more details, see Supplemental Methods.

### Primary samples

CML samples were leukapheresis products isolated from individuals with chronic phase CML at the time of diagnosis prior to TKI treatment. Non-CML samples were surplus cells collected from femoral-head BM, surgically removed from patients undergoing hip replacement or leukapheresis products from individuals with non-myeloid Ph^−^ haematological disorders. CD34^+^ cells were isolated using the CD34 MicroBead Kit or CliniMACS (both Miltenyi Biotec).

### Cell culture

All cultures were performed at 37 °C in a 5% CO_2_ incubator (Eppendorf). For more details, see Supplemental Methods.

### Stem cell and differentiation analysis in CD34^+^ CML cells

CD34^+^ CML cells were stained with 1 μM CellTrace Violet (CellTrace Violet Cell Proliferation Kit, Life Technologies) in PBS for 30 min at 37 °C. The reaction was quenched by adding cell culture medium containing 10% FBS. Cells were then washed and re-suspended in SFM supplemented with PGF cocktail and treated as indicated in figure legends. After 3 or 6 days, cells were stained with anti-human CD34-APC (BD Biosciences), anti-human CD38-PerCP (BioLegend) and anti-human CD133-PE (Miltenyi Biotec). For detection of differentiation markers, cells were stained with anti-human CD71-PE, anti-human CD11b-PE-Cy7 and anti-human CD14-APC-Cy7 (all from BioLegend) followed by flow cytometry analysis (FACSVerse^TM^ Flow Cytometer, BD Biosciences). Data analysis was performed using FlowJo 7.6.5 software.

### Cell cycle analysis

CD34^+^ CML cells were fixed with 70% ethanol in PBS following 3 days treatment with the indicated drugs (see figure legends). Permeabilization was performed as previously described. [4] For more details, see Supplemental Methods.

### Dual-fusion interphase fluorescence in situ hybridization (D-FISH)

See Supplemental Methods.

### In vitro treatments, CFC and LTC-IC assays

CFC and LTC-IC was performed as previously described [4]. For more details, see Supplemental Methods.

### Immunofluorescence of bone sections, LC3 puncta and RFP-GFP-LC3 detection

See Supplemental Methods.

### Western blot analysis, RNA extraction and quantitative PCR

See Supplemental Methods.

### Statistics and study approval

Significance is indicated as follows: *p < 0.05; **p < 0.01; ***p < 0.001. For more details, see Supplemental Methods.

## Results

### Primitive CML cells have high levels of basal autophagy in vivo

**Fig. 1:**
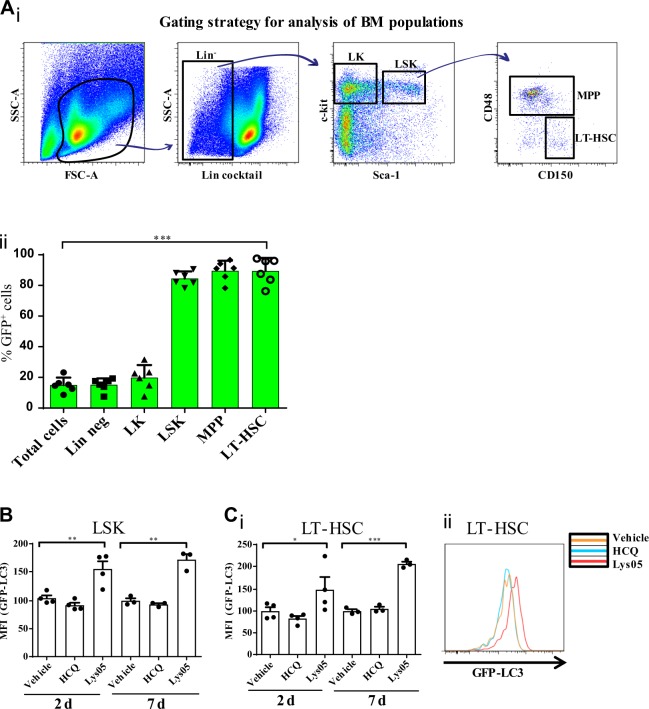
Autophagy levels in LT-HSCs following in vivo treatment with HCQ or Lys05. **a** Representative gating strategy used for analysis of the different BM populations by flow cytometry (i). Percentage of GFP^+^ cells in the following BM populations of leukemic mice (*n* = 6); linage negative (Lin^−^); Lin^-^Sca-1^-^c-kit^+^ (LK); Lin^-^Sca^+^c-kit^+^ (LSK); LSK/CD48^+^CD150^-^ (MPP), LSK/CD48^-^CD150^+^ (LT-HSC) (ii). **b**, **c** Levels of GFP-LC3 in LSK (**b**) and LT-HSC (**c** i) following in vivo treatment with vehicle (PBS), HCQ or Lys05 for 2 days (*n* = 4/arm) or 7 days (*n* = 3/arm). Results are shown as mean fluorescence intensity (MFI) and relative to vehicle-treated mice. Representative histogram showing the shift in GFP-LC3 levels in LT-HSCs following Lys05 treatment (c ii). Error bars represent ±SEM

To measure basal autophagy levels in CML cells in vivo we crossed a previously described transgenic mouse model of CML (*Scl-tTa-BCR-ABL*) [21] with a mouse expressing the autophagy marker LC3 fused to GFP [22]. After removal of tetracycline from the drinking water, *BCR-ABL* expression is induced (tet-off system) in the HSC population and mice develop a CML-like disease, characterized by splenomegaly, myeloid hyperplasia and a reduction in erythrocytes and B-cells (Figure S1A-B). An increase in fluorescence in cells expressing GFP-LC3 indicates an accumulation of autophagic vesicles [24]. Analysis of the percentage of GFP^+^ cells within different leukemic cell populations, isolated from the BM of *Scl-tTa-BCR-ABL/GFP-LC3* mice, revealed that whereas only 15–20% of the cells were GFP^+^ in the lineage negative (Lin^−^) and Lin^−^c-kit^+^ (LK) populations, 85–90% of the cells in the Lin^−^Sca-1^+^c-kit^+^ (LSK), multipotent progenitors (MPP) and the long-term (LT)-HSC compartments were GFP^+^, reflecting a significantly higher content of autophagosomes in primitive populations (Fig. 1a). Similar results were obtained when the percentage of GFP^+^ cells within these populations was analysed in BM of non-leukemic mice (Figure S1C). To confirm active autophagic flux in these primitive cells, LSK cells were isolated from leukemic mice and cultured without cytokines to induce autophagy. In line with previous studies performed in normal haematopoietic cells, autophagy induction was linked to increased degradation of GFP-LC3 and therefore a reduction in GFP-LC3 levels [25], which was reverted by HCQ-mediated autophagy inhibition (Figure S1D-F). A similar effect was observed when LC3-II levels were measured by flow cytometry following TKI treatment (Figure S1G). Importantly, comparison of autophagy flux in LSK cells from leukemic and non-leukemic mice revealed increased flux in primitive leukemic cells compared with their normal counterparts (shown by increase in GFP-LC3 levels following Lys05-mediated autophagy inhibition; Figure S1H).

### Lys05 inhibits autophagy in LT-HSCs in vivo and patient-derived CML CD34^+^ cells

Recent Phase I studies indicate that more potent autophagy inhibitors are required to consistently inhibit autophagy in cancer patients [12–16]. To assess whether autophagy inhibition can be achieved in LSCs in vivo, we treated leukemic mice with HCQ or Lys05, followed by BM extraction and GFP-LC3 detection in LSK cells and LT-HSCs. Notably, HCQ treatment did not affect GFP-LC3 levels, whereas an increase in fluorescence was observed in LSK cells and LT-HSCs purified from Lys05 treated mice, indicating potent autophagy inhibition with the latter agent (Figs. 1b-c). To confirm autophagy inhibition using a separate autophagy marker, we treated leukemic mice and quantified the accumulation of the autophagy cargo receptor sequestosome 1 (SQSTM1/p62) in BM cells; when autophagic flux is blocked, SQSTM1/p62 accumulates [24]. Immunofluorescence staining of BM sections demonstrated that Lys05, but not HCQ treatment, produced significantly higher levels of SQSTM1/p62 accumulation compared to controls in leukemic cells (Figure S1I-J).

**Fig. 2:**
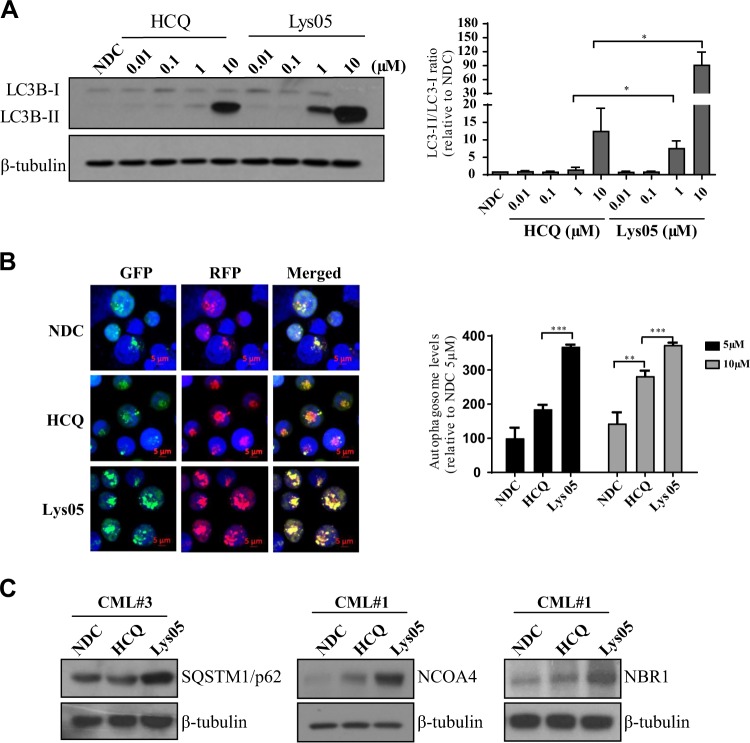
Autophagy levels in leukemic cells, including CD34^+^ cells from CML patients, following in vitro treatment with HCQ or Lys05. **a** Representative blot and quantification analysis (*n* = 3) showing the levels of LC3-I/II in K652 cells following 4 h treatment with vehicle (NDC), HCQ or Lys05 at increasing concentrations from 0.01 to 10 µM. β-tubulin was used as loading control. The ratio between LC3-II and LC3-I was calculated with the densitometry values for each of the conditions and normalized to the values of the correspondent β-tubulin. Results are represented as fold change to NDC. Error bars represent ±SEM. **b** Representative confocal fluorescent images of K562 cells expressing mRFP-GFP-LC3 following 4 h treatment with vehicle (NDC), HCQ (10 µM), Lys05 (10 µM). Quantification of autophagosomes levels were calculated using the co-localization coefficient between red (RFP) and green (GFP). Error bars represent ±SD. **c** Representative blots showing the expression levels of SQSTM1/p62, NCOA4 and NBR1 on CD34^+^ cells. *n* = 3 individual patient samples were assessed. Membranes were re-probed with an anti-β-tubulin antibody as loading control

Comparing the levels of autophagy inhibition between Lys05 and HCQ in human CML cells, K562 cells were treated with both inhibitors at increasing concentrations. Following 4 hours (h) treatment, a more significant increase in membrane-bound LC3B- phosphatidylethanolamine conjugate (LC3B-II) was observed with Lys05 at 1 µM and 10 µM concentration, indicating increased accumulation of autophagosomes (Fig. 2a). To precisely assess autophagy flux, we generated a K562 cell line stably expressing fluorescence-tagged human LC3B (mRFP-GFP-LC3) that enables different stages of autophagy to be visualized by fluorescence microscopy [26]. The appearance of red/green puncta (yellow when merged) indicate autophagosomes, and as the acidic conditions in the lysosomes quench the GFP fluorescence, “red only” puncta indicate autolysosomes. Autophagic flux can be inhibited by lysosomotropic drugs, which prevent the fusion of autophagosomes and lysosomes, leading to build-up of yellow fluorescence. Whereas 5 µM HCQ treatment failed to eliminate “red only” puncta, Lys05 treatment led to a significant accumulation of yellow fluorescence (*p* < 0.001), indicating a complete block in autophagy flow (Fig. 2b). Similar results were observed in a second human cell line—KCL22, demonstrating that the increased potency of Lys05 is not restricted to K562 cells (Figure S2A-B).

To evaluate autophagy inhibition in patient-derived material, we next measured autophagy inhibition following drug treatment in stem cell-enriched (CD34^+^) cells, isolated from individuals with chronic phase CML. Since previous pharmacokinetic studies demonstrated that the maximal achievable concentration of HCQ in plasma in cancer patients, treated with non-toxic 800 mg/day dose, is ~2000 ng/mL (~5 µM) [12–16] we used 3–5 µM drug concentration of each drug in the following experiments. After treatment with a single dose of either HCQ or Lys05, immunofluorescence assays showed accumulation of LC3 puncta after Lys05 treatment (Figure S2C). Analysis of additional autophagy substrates by western blot, SQSTM1/p62, neighbour of BRCA1 gene 1 (NBR1) [27] and nuclear receptor coactivator 4 (NCOA4) [20, 28], revealed a consistent increase after Lys05 treatment (Fig. 2c), confirming that Lys05 inhibits autophagy in primary CML progenitor cells more consistently than clinically achievable concentrations of HCQ.

### Lys05-mediated autophagy inhibition reduces the numbers of LSCs in vivo and in vitro

**Fig. 3:**
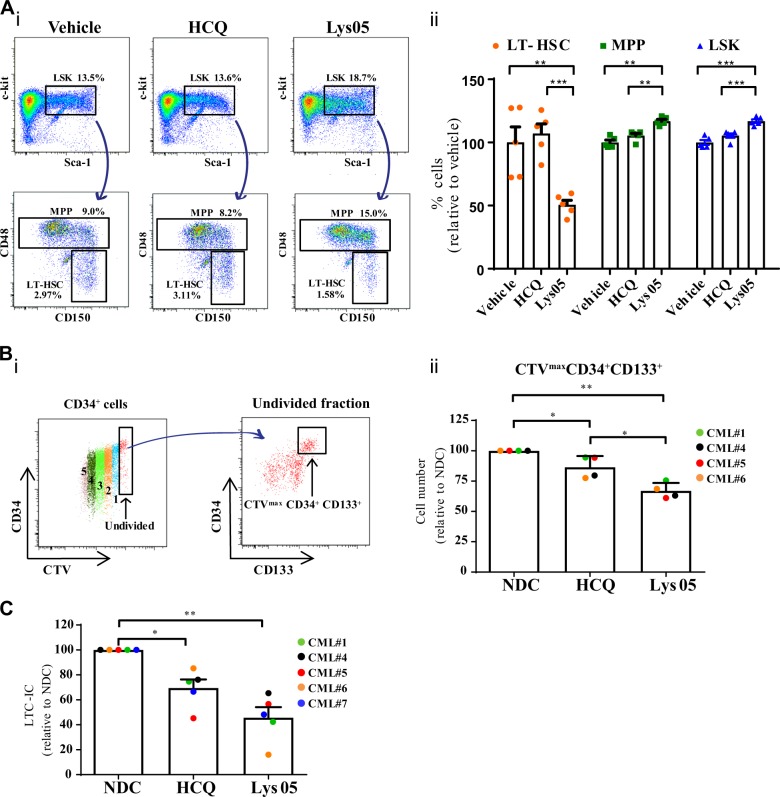
Effects of autophagy inhibition on LSC viability. **a** Representative dot plots showing the LSK, MPP and LT-HSC populations in the BM of leukemic mice following in vivo treatment (i). Percentage of LT-HSCs, MPPs and LSK cells in the BM of leukemic mice after 2 days of in vivo treatment with vehicle (PBS, *n* = 5), HCQ (*n* = 5) or Lys05 (*n* = 5). Results are represented relative to vehicle-treated mice. Error bars represent ±SEM (ii). **b** Representative plots obtained from CellTrace Violet-stained (CTV) CD34^+^ CML cells cultured with SFM + PGF for 3 days (i). Left plot shows the number of divisions (shown in different colours) pointing with an arrow at the undivided fraction (CTV^max^). Right plot shows the CD34^+^CD133^+^ population gated on the undivided fraction (CTV^max^CD34^+^CD133^+^). Number of CTV^max^CD34^+^CD133^+^ cells after 3 days of treatment with vehicle (NDC), HCQ (5 µM) or Lys05 (5 µM). *n* = 4 individual patient samples (ii). (**c**) Total number of LSC-derived colonies measured by LTC-IC assay of CD34^+^ CML cells following treatment with vehicle (NDC), HCQ (5 µM) and Lys05 (5 µM). *n* = 5 individual patient samples. Results are represented relative to NDC

We next analysed the cellular effects on different BM progenitor populations of leukemic mice. Intriguingly, Lys05-treated mice showed a 50% reduction in the most primitive LT-HSC population (*p* < 0.01), with no significant effect seen in HCQ-treated mice (Fig. 3a; S3A). This reduction in LT-HSCs was accompanied by a significant expansion in the MPP (*p* < 0.01) and LSK (*p* < 0.001) compartments, suggesting Lys05 targets LT-HSCs in leukemic mice by promoting their maturation. In contrast, no significant effect was seen in Lys05-treated non-leukemic mice (Figure S3B).

As human CD34^+^ cells are a heterogeneous population and genuine CML LSCs represent a small fraction of total CD34^+^ cells, we have previously focused on those CD34^+^ cells that remain quiescent in culture [4, 29]. Therefore, we investigated the effects of HCQ and Lys05 in more primitive populations by combining assessment of CD34 and CD133 [30] expression, with the cell division tracer Cell Trace Violet (CTV), which allows multiplexing due to the limited spectral overlap with other fluorescent probes. Although both compounds significantly reduced the number of the undivided cells with high CD34 and CD133 expression (CTV^max^CD34^+^CD133^+^), the effect of 5 µM Lys05 was more compelling, promoting a 33% reduction after 3 days treatment (*p* < 0.01, Fig. 3b). Additionally, we measured the number of cells within another well-defined primitive population, CD34^+^CD38^−^. Although both 3 and 5 µM HCQ treatment significantly decreased the cell number (*p* < 0.05), treatment with equimolar concentrations of Lys05 reduced the number of CD34^+^CD38^−^ cells to a greater extent (Figure S3C). We next performed long-term culture initiating cell (LTC-IC) assays using CD34^+^ cells. This most stringent in vitro stem cell assay demonstrated that, while HCQ had a moderate effect (*p* < 0.05), Lys05 treatment reduced the number of colonies by 54% (*p* < 0.01) when compared to the untreated control (Fig. 3c), and by 90% following 6 days treatment (Figure S3D).

### Lys05 promotes loss of quiescence and induces maturation of human CML cells

**Fig. 4:**
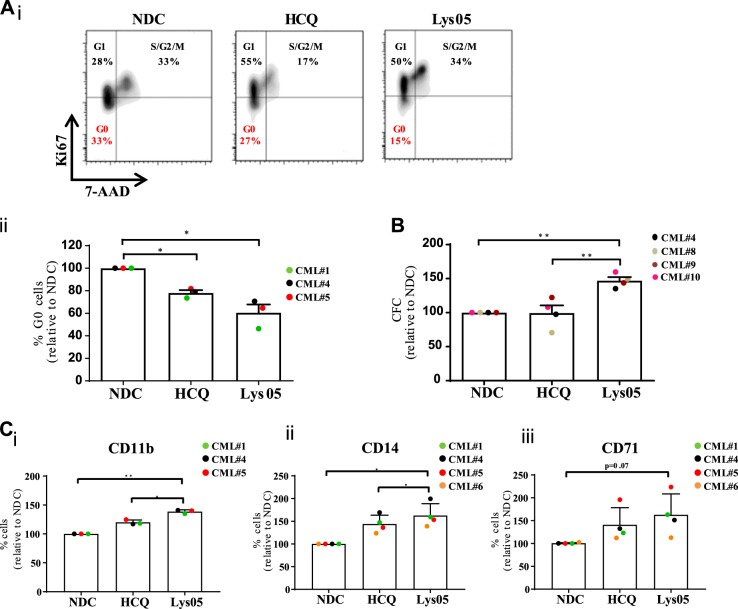
In vitro effects of autophagy inhibition on cell cycle and differentiation. **a** Representative cell density plots showing cell cycle analysis by co-staining with Ki67/7^−^AAD. The percentage of quiescent (Ki67^−^) cells is highlighted in red (i). Percentage of Ki67^-^ CD34^+^ CML cells following 3 days treatment with vehicle (NDC), HCQ (3 µM) and Lys05 (3 µM) (ii). **b** Total number of colonies obtained from CFCs following 3 days drug treatment of CD34^+^ CML cells with vehicle (NDC), HCQ (3 µM) and Lys05 (3 µM). *n* = 4 patient samples. **c** Levels of CD11b (i; *n* = 3), CD14 (ii; *n* = 4) and CD71 (iii, *n* = 4) on CD34^+^ CML cells following 3 days treatment with vehicle (NDC), HCQ (5 µM) or Lys05 (5 µM). Results are represented as percentage of cells relative to NDC. Error bars represent ±SEM between patient samples

Given our in vivo data which suggested induced maturation of LT-HSCs following Lys05 treatment in leukemic mice (Fig. 3a; S3A), we hypothesized that the decrease in LTC-IC number could be explained by Lys05 driving the LSCs out of quiescence, into a more proliferative phenotype. Indeed, cell cycle analysis using the proliferation marker Ki67, combined with DNA staining using 7-AAD, showed that the G0 fraction (Ki67^low^7-AAD^low^) was decreased after treatment with either compound alone (*p* < 0.05), with 39% reduction on average observed following Lys05 treatment (Fig. 4a). Similar results were obtained in LSK cells isolated from leukemic mice (Figure S3E). This correlated with an increase in colony-forming cell (CFC) number following Lys05 treatment (Fig. 4b). To assess whether this observation was linked to cellular differentiation, we measured the expression of several mature myeloid surface markers. Levels of the granulocyte/monocyte marker CD11b, the macrophage/neutrophil marker CD14 and the erythroid marker CD71 were increased following autophagy inhibition, with greater effects observed in Lys05-treated cells (Fig. 4c). These data further indicate that autophagy inhibition induces loss of quiescence and drives LSCs into differentiation.

To demonstrate that the phenotypic effects of Lys05 treatment on leukemic mice were primarily due to autophagy inhibition, we generated an autophagy deficient CML model by crossing the previously described *Atg7*^*flox/flox*^*:Mx-Cre* conditional knockout mice [23] with the *Scl-tTa-BCR-ABL* model. Following removal of tetracycline, mice were injected with polyinosinic-polycytidylic acid (pIpC) to induce a recombinase-dependent deletion of the essential autophagy gene *Atg7*. As expected, a significant reduction in *Atg7* mRNA levels was achieved, which correlated with a significant accumulation of SQSTM1/p62 staining in BM cells, indicating an autophagy deficiency (*p* < 0.01, Figure S4A-C). Although the analysis of leukemic BM cells revealed no significant difference in LT-HSCs, there was a clear increase in the relative proportions of MPP (*p* < 0.001) and LSK cells (*p* < 0.01) resembling the progenitor expansion observed in Lys05-treated mice (Figure S4D). Similar effects were seen in non-leukemic LT-HSCs, MPP and LSK cells (Figure S4E). Overall, these data suggest that Lys05 achieves autophagy inhibition in LSCs and promotes differentiation similar to genetic autophagy deficiency.

### Novel autophagy inhibitors sensitize patient-derived LSCs to TKI treatment in vitro and in vivo

**Fig. 5:**
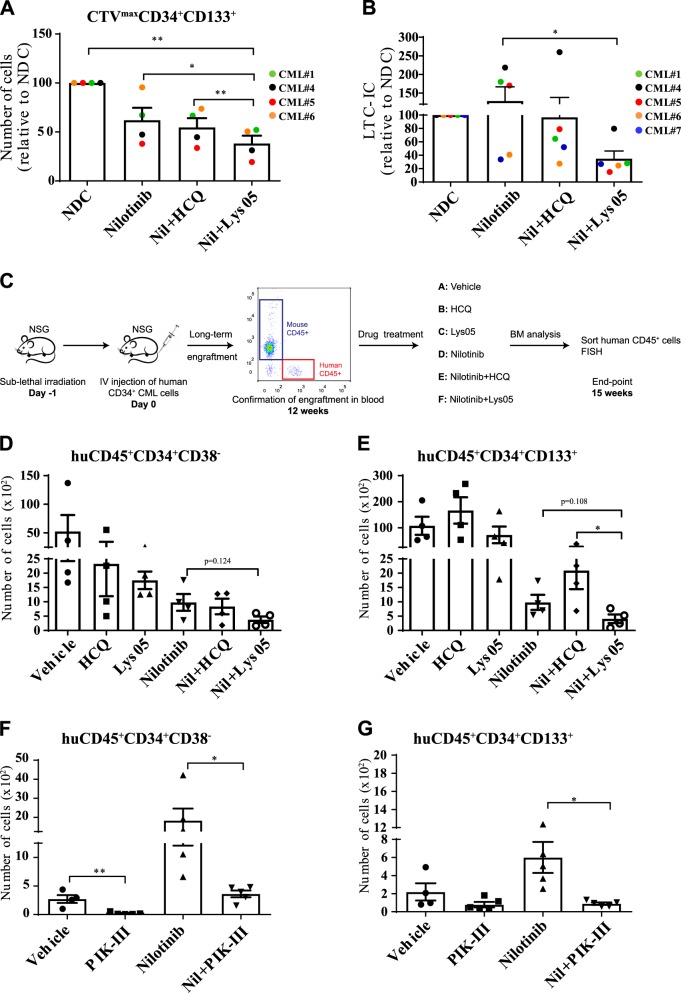
Effects of combined inhibition of autophagy and BCR-ABL on LSCs in vitro and in vivo. **a** Number of CTV^max^ CD34^+^ CD133^+^ cells following 3 days of treatment with vehicle (NDC), nilotinib (2 µM), or combinations of nilotinib with either HCQ (5 µM) or Lys05 (5 µM). *n* = 4 patient samples. **b** Total number of LSC-derived colonies measured by LTC-IC assay of CD34^+^ CML cells following treatment for 3 days with vehicle (NDC), nilotinib (2 µM), or combinations of Nil with either HCQ (5 µM) or Lys05 (5 µM). *n* = 5 patient samples. Results are represented relative to NDC. Error bars represent ±SEM. **c** Schematic representation showing the experimental design for in vivo treatment of NSG mice with vehicle (*n* = 4), HCQ (*n* = 4), Lys05 (*n* = 4), nilotinib (*n* = 4) and combinations of nilotinib with each of the autophagy inhibitors (*n* = 4 for each combination). **d**, **e** Absolute cell number of human CD45^+^CD34^+^CD38^–^ (**d**), CD45^+^CD34^+^CD133^+^ (**e**) from the BM of each mouse following 3 weeks of in vivo treatment. **f**, **g** Absolute cell number of human CD34^+^CD38^–^ (G) and CD34^+^CD133^+^ (**h**) extracted from the BM of NSG mice 16 weeks post-transplant. Before transplant, CD34^+^ cells were treated ex vivo for 48 h with vehicle, PIK-III (5 µM), nilotinib (2 µM) or combination of PIK-III with nilotinib. Error bars represent ±SEM

To date it is unknown whether clinically achievable concentrations of HCQ sensitize CML LSCs to TKI treatment, and if this combination is effective in vivo. To test the effects of combined inhibition of autophagy and BCR-ABL kinase activity on LSCs we first measured the number of CTV^max^CD34^+^CD133^+^ cells after treatment with either HCQ or Lys05 in the presence of the second generation TKI nilotinib, which is at least 10-fold more potent than imatinib [31]. Figure 5a shows that, whereas 5 µM HCQ in combination with nilotinib did not have any additional effects compared to nilotinib alone, 5 µM Lys05 combined with nilotinib showed a significant reduction in number of CTV^max^CD34^+^CD133^+^ cells compared to the TKI alone (*p* < 0.05, Fig. 5a). This additive effect was not as noticeable in bulk CD34^+^ cells, indicating that the drug combination displayed selectivity towards more primitive populations (Figure S5A). LTC-IC assays confirmed these results, demonstrating a reduction in LSC survival after combined treatment with Lys05 and nilotinib, compared to TKI alone (Fig. 5b; S5B). Real-Time quantitative PCR of individual colonies for BCR-ABL expression confirmed the presence of the Philadelphia (Ph) chromosome in 100% of the colonies (Figure S5C).

To test the in vivo effect of these combinations on the most primitive human LSCs, we used a robust PDX model of human CML. Sub-lethally irradiated immunocompromised NOD-SCID-γc^−/−^ (NSG) mice were transplanted with CD34^+^ CML cells. Twelve weeks following transplantation, engraftment was assessed by measuring the expression of human CD45 in peripheral leukocytes in the blood. After ensuring equivalent and sufficient engraftment, mice were treated daily with HCQ and Lys05 as single agents and in combination with nilotinib (Fig. 5c). In all experimental arms, no changes in mouse or spleen weight were observed indicating excellent tolerability (Figure S5D-E). Three weeks of in vivo treatment with nilotinib reduced the absolute number of CML-derived CD45^+^ leukocytes (Figure S5F) and engrafted CD45^+^CD34^+^CD38^−^and CD45^+^CD34^+^CD133^+^ human cells within the BM (Figs. 5d-e), but the proportion of CML-derived leukocytes in spleen was unaffected (Figure S5G). Of clinical relevance, while the combination of HCQ and nilotinib had modest or no additional effect compared to nilotinib as a single agent, Lys05 treatment enhanced the effects of the TKI by reducing the levels of human CD45^+^ cells in BM and spleen (*p* = 0.05 and *p* < 0.01 respectively, Figure S5F-G). Strikingly, although the combination of Lys05 and nilotinib were not statically different than nilotinib alone, the combination of Lys05 and nilotinib eliminated 93% of CD45^+^CD34^+^CD38^−^and 96% of CD45^+^CD34^+^CD133^+^ engrafted cells within the BM (Figs. 5d-e). Fluorescent in situ hybridization, performed on sorted human CD45^+^ cells from the BM confirmed the presence of the BCR-ABL fusion gene in over 95% of the cells (Figure S5H).

Encouraged by our results with Lys05, we further investigated whether a more selective autophagy inhibitor could also target LSCs when used in combination with nilotinib. To address this we used PIK-III, a recently developed inhibitor of the lipid kinase VPS34 [20]. Treatment with PIK-III led to the accumulation of the autophagy substrates, NBR1, NCOA4 and SQSTM1/p62 confirming inhibition of autophagy in CD34^+^ CML cells (Figure S6A-B). Furthermore, PIK-III treatment inhibited TKI-induced autophagy when used in combination with nilotinib (Figure S6C). We next examined the effect on the primitive CTV^max^CD34^+^CD133^+^ population. This revealed that 69% of CTV^max^CD34^+^CD133^+^ cells were eliminated following PIK-III alone (*p* < 0.01, Figure S6D). Moreover, 90% of CTV^max^CD34^+^CD133^+^ cells were eliminated following the combination of PIK-III and nilotinib (*p* < 0.001). Since PIK-III is not suitable for in vivo treatment [20], to examine the effect on engraftment of primitive LSCs, CD34^+^ CML cells were treated for 48 h with PIK-III, nilotinib or the combination, and transplanted into NSG mice with the engraftment of human Ph^+^ cells measured after 16 weeks. In agreement with our previous work, in vitro TKI treatment leads to enrichment of primitive cells [4, 29, 32]. The reduction in engrafted CD45^+^CD34^+^ CML cells following single PIK-III treatment, including primitive CD45^+^CD34^+^CD38^−^and CD45^+^CD34^+^CD133^+^ populations, confirmed the essential role of autophagy in the maintenance of the LSC pool (Figs. 5f-g; S6E). Importantly, comparison of the combination treatment with single TKI treatment revealed a decrease in the number of CML LSCs in the combination arm, further confirming the importance of autophagy in TKI-treated quiescent LSCs.

To test the effect of second generation autophagy inhibitors on normal blood cells, CD34^+^ cells derived from separate donors were treated with HCQ, Lys05 or the combination of PIK-III and nilotinib, and compared with the cytotoxicity of 20 nM bortezomib and 10 nM omacetaxine treatment. This revealed that while bortezomib and omacetaxine substantially affected the CFC potential of normal progenitor cells, the effect of Lys05 and PIK-III-mediated autophagy inhibition had only minimal effect (Figure S6F-G). Since neither Lys05 nor PIK-III promoted an increase in the number of CFCs, our results also suggested that the myeloid expansion seen following autophagy inhibition in CML cells (Figs. 4b-c) was selective for Ph^+^ cells.

### Combination of TKI and Lys05 eliminates transplantable LT-HSCs

**Fig. 6:**
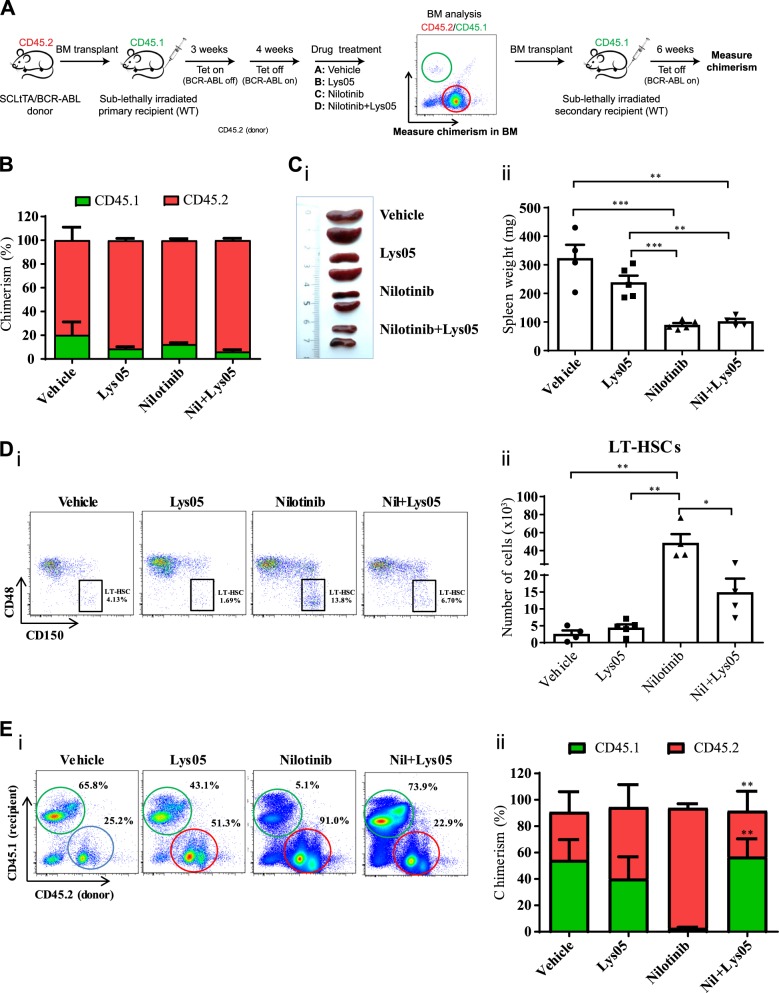
Targeting repopulating cells in Scl-tTa-BCR-ABL mice by combining Lys05 with nilotinib. **a** Schematic diagram of the experimental design. BM cells obtained from *Scl-tTa-BCR-ABL* CD45.2 mice were transplanted (2 × 10^6^cells/mouse) into WT CD45.1 sub-lethally irradiated recipients (2 × 4.25 Gy, 3 h a part). 3 weeks post-transplant, tetracycline was removed and after 4 weeks, the mice were randomly separated in 4 experimental arms as indicated. Mice were treated for 21 days with vehicle (*n* = 4); Lys05 (*n* = 5), nilotinib (*n* = 5) or combination of Lys05 and nilotinib (*n* = 4) followed by analysis of the BM and spleens. BM cells were transplanted into new CD45.1 WT recipients (3 × 10^6^ cells/mouse) and the mice were left off tetracycline for 6 weeks before analysis of the BM. **b** CD45.1 and CD45.2 cells shown as percentage of total BM cells in primary recipients following 21 days of in vivo treatment with vehicles (*n* = 4); Lys05 (*n* = 5), nilotinib (*n* = 5) or combination of Lys05 and nilotinib (*n* = 4). **c** Representative photograph of the spleens collected from primary transplanted mice after the treatment (i). Mouse spleen weight from each experimental arm (ii). **d** Representative plots showing the percentage of CD45.2^+^ LT-HSCs in the BM of treated mice (i). Absolute number of CD45.2^+^ LT-HSCs in the BM of leukemic mice following 21 days of treatment (ii). **e** Representative plots showing the percentage of CD45.1 and CD45.2 cells in the BM of secondary recipients 6 weeks post-transplant (i). Percentage of CD45.1 and CD45.2 cells in BM of secondary recipients from all experimental arms (vehicle, *n* = 5; Lys05, *n* = 5; nilotinib, *n* = 6; combination of Lys05 and nilotinib, *n* = 6) measured at week 6 post-transplant (ii). Error bars represent ±SEM

To further study the selectivity and the functional effect of combining Lys05 with TKI on transplantable LSCs in vivo, we used the *Scl-tTa-BCR-ABL* model, which allows discrimination of host and donor cells in a transplantation setting using the CD45.1/45.2 system. First, we transplanted BM cells from CD45.2 *Scl-tTa–BCR-ABL* donor mice into sub-lethally irradiated CD45.1 wild type (WT) recipients (*n* = 18). Following tetracycline removal, induction of BCR-ABL expression and evidence of CML-like disease, four cohorts of mice were treated with vehicle, Lys05, nilotinib or the combination for three weeks (Fig. 6a). As expected, although the vast majority of the cells in the BM were of donor origin (CD45.2) (Fig. 6b), the leukemic burden was reduced in nilotinib-treated mice, evidenced by reduction in splenomegaly and absolute numbers of CD45.2^+^ donor cells in the spleen (Fig. 6c; S7A). As previously reported, relatively low numbers of transplanted LT-HSCs were visible in the host BM of leukemic mice due to the rapid differentiation induced by BCR-ABL expression [33] (Fig. 6d). Whereas the anti-proliferative effect of nilotinib resulted in a significant accumulation of CD45.2 LT-HSCs in treated recipient mice (*p* < 0.01), the combination with Lys05 treatment reduced this effect by 69% (*p* < 0.05), mirroring the efficacy of PIK-III in eliminating LSCs when combined with TKI treatment in the xenograft model (compare Figs. 5f-g with 6Dii). Single colony PCR confirmed BCR-ABL expression in surviving CD45.2 LSK cells (Figure S7B). To assess whether the surviving cells possessed repopulation potential, BM cells from each separate cohort were pooled and 3 × 10^6^ cells transplanted into secondary CD45.1 WT recipients. Following 6 weeks of disease development, analysis of the chimerism between CD45.1 and CD45.2 cells in the BM of secondary recipients was assessed. This revealed that mice transplanted with BM cells from vehicle and Lys05 treated mice showed similar CD45.1/CD45.2 ratio for vehicle (54 ± 15/55 ± 15) and for Lys05 (40 ± 16/54 ± 17), whereas BM from the nilotinib treated mice repopulated the secondary recipients almost exclusively with CD45.2 cells (Fig. 6e). This was in line with the increased number of LT-HSCs in nilotinib treated primary recipients (Fig. 6). Strikingly, 4 out of 6 mice receiving BM from the combination cohort had a higher ratio of CD45.1 non^−^leukemic cells in BM of secondary recipients, indicating that the addition of Lys05 to nilotinib selectively targeted leukemic cells with repopulation capacity over normal LT-HSCs (Fig. 6e).

## Discussion

Although studies have described a role for autophagy in normal haematopoiesis [25, 34–36], the consequences of modulating this process in a leukemic microenvironment is currently unknown. Here, using in vivo CML models, we show for the first time that primitive leukemic cells have higher autophagy levels than more differentiated cells. This observation highlights the importance of studying the effects of autophagy inhibition in the most clinically relevant population, known to be resistant to the standard of care for CML patients.

We use a double transgenic model of CML, where BCR-ABL expression is induced in stem cells [21], and demonstrate that Lys05 treatment consistently inhibits autophagy in CML LSCs in vivo, while HCQ approximating clinically achievable concentrations fails to do so. In terms of molarity, the dose of HCQ used in vivo (32 mg/kg/day; 73.74 nmols/g), corresponds to double the concentration of Lys05 used (20 mg/kg/day; 36.37 nmols/g), which is lower than the Lys05 dosage used in previous studies [17, 37], underlining the improved potency of Lys05 in inhibiting autophagy in LSCs in vivo. Interestingly, we found that an active autophagic flux is essential for the maintenance of undifferentiated quiescent LSCs, since potent autophagy inhibition with Lys05 led to a rapid reduction of LT-HSCs in vivo, followed by an increase in progenitor cells (represented by the MPP and LSK compartments). These results are in line with our in vitro experiments using CD34^+^ cells derived from CML patients, where Lys05 treatment decreases the number of LTC-ICs and increases the CFCs derived from progenitor cells. Notably, these effects were either absent or lower following in vivo or in vitro treatment with HCQ at clinical achievable concentrations.

In terms of the role autophagy plays in preventing maturation of haematopoietic cells, our findings are in agreement with results obtained by Ho and colleagues, which showed that genetic inhibition of autophagy by ATG12 deletion leads to an expansion of myeloid progenitor cells [34]. In addition, conditional deletion of ATG7 or FIP200 in the haematopoietic system triggers myeloid differentiation and expansion of LSK cells, followed by a reduction of fetal HSCs [35] or HSCs from young (7-week-old) mice [36]. In our work, we generated a double inducible system, which allowed simultaneous ATG7 deletion and BCR-ABL induction in adult HSCs. When comparing autophagy deficient leukemic (and non-leukemic) mice with autophagy competent mice, leukemic ATG7^−/−^ mice showed an expansion of the LSK fraction (which was similar to Lys05-treated leukemic mice), although no significant differences in LT-HSCs were observed between the two phenotypes. This could be explained by the rapid development of leukemia we observed in ATG7^+/+^ compared to ATG7^−/−^ mice; due to the differentiation of LT-HSCs following BCR-ABL expression previously reported in this model [38], a more advanced stage of disease in the autophagy competent mice would mask the decrease of LT-HSCs following ATG7 deletion. Further studies are required to assess the role of autophagy in different stages of the disease and to delineate cell type specific responses to autophagy inhibition, which will lead to deeper understanding of the biological implications of autophagy in leukemia development and progression. In this regard, the status of p53 should be considered, since several reports have indicated that the tumour suppressor is essential for the role autophagy plays in tumour initiation and progression in other types of cancer [39, 40]. Nevertheless, the expansion of progenitor cells observed in our model, reinforced by results in normal haematopoiesis, strongly support that the Lys05-mediated effect in LSCs is due to autophagy inhibition.

A possible mechanism for the differentiation of primitive cells following Lys05 treatment is an increase in mitochondria-derived reactive oxygen species (ROS). We and others have previously linked autophagy deficiency in leukemic cells with an increase in mitochondria content and elevated oxidative phosphorylation [11, 34]. It is possible that the increase in oxidative phosphorylation could lead to ROS leakage from the electron transport chain. In fact, increased ROS caused by deletion of FoxO, a transcriptional factor that regulates the expression of antioxidant proteins, leads to a myeloid expansion [41], resembling the increase in myeloid progenitor cells we observed following in vivo treatment with Lys05.

Recent Phase I dose-escalation clinical trials, where maximum tolerated dose and safety of HCQ was evaluated, have indicated that autophagy inhibition remains a promising strategy for improving the efficacy of cancer therapy. However, these studies also revealed that dose-limiting toxicity prevents escalation to a high enough dose of HCQ to reach 10 µM concentration in plasma [12–16]. Therefore, HCQ-mediated autophagy inhibition is not consistently achieved in cancer patients, emphasizing the need to investigate more potent second generation autophagy inhibitors in robust pre-clinical models. Both in vitro studies and the robust PDX model showed that Lys05 and PIK-III, but not HCQ, enhance the effects of TKI on the most primitive cells. Additionally we demonstrate that the combination of Lys05 and nilotinib can eliminate cells that are able to regenerate transplantable disease, while the treatment with TKI as single agent enriched for these cells. This is critical since LT-HSCs have shown heterogeneity in leukemia-initiating capacity, suggesting that only a sub-fraction of long-term engrafting cells has LSC capacity [42].

Finally, CD34^+^ normal cells were only modestly affected by treatment with PIK-III or Lys05 compared to CD34^+^ CML cells, suggesting a potential therapeutic window for autophagy inhibition as a CML therapy. Therefore, given that the presence of LSCs after TKI treatment has been associated with disease relapse [43], our results provide a strong rationale for considering second generation autophagy inhibitors as a potential clinical option for CML patients with minimal residual disease.

## Electronic supplementary material

Supplementary Figures + Figure Legends

Supplementary Methods

## References

[1] Druker BJ, Guilhot F, O’Brien SG, Gathmann I, Kantarjian H, Gattermann N (2006). 5-year follow-up of patients receiving imatinib for chronic myeloid leukemia. N Engl J Med.

[2] Holyoake TL, Vetrie D (2017). The chronic myeloid leukemia stem cell: stemming the tide of persistence. Blood.

[3] Corbin AS, Agarwal A, Loriaux M, Cortes J, Deininger MW, Druker BJ (2011). Human chronic myeloid leukemia stem cells are insensitive to imatinib despite inhibition of BCR-ABL activity. J Clin Invest.

[4] Hamilton A, Helgason GV, Schemionek M, Zhang B, Myssina S, Allan EK (2012). Chronic myeloid leukemia stem cells are not dependent on Bcr-Abl kinase activity for their survival. Blood.

[5] Holyoake TL, Helgason GV (2015). Do we need more drugs for chronic myeloid leukemia?. Immunol Rev.

[6] Karsli-Uzunbas G, Guo JY, Price S, Teng X, Laddha SV, Khor S (2014). Autophagy is required for glucose homeostasis and lung tumor maintenance. Cancer Discov.

[7] Guo JY, Teng X, Laddha SV, Ma S, Van Nostrand SC, Yang Y (2016). Autophagy provides metabolic substrates to maintain energy charge and nucleotide pools in Ras-driven lung cancer cells. Genes Dev.

[8] Kuma A, Hatano M, Matsui M, Yamamoto A, Nakaya H, Yoshimori T (2004). The role of autophagy during the early neonatal starvation period. Nature.

[9] Rebecca VW, Amaravadi RK (2016). Emerging strategies to effectively target autophagy in cancer. Oncogene.

[10] Bellodi C, Lidonnici MR, Hamilton A, Helgason GV, Soliera AR, Ronchetti M (2009). Targeting autophagy potentiates tyrosine kinase inhibitor-induced cell death in Philadelphia chromosome-positive cells, including primary CML stem cells. J Clin Invest.

[11] Karvela M, Baquero P, Kuntz EM, Mukhopadhyay A, Mitchell R, Allan EK (2016). ATG7 regulates energy metabolism, differentiation and survival of Philadelphia-chromosome-positive cells. Autophagy.

[12] Mahalingam D, Mita M, Sarantopoulos J, Wood L, Amaravadi RK, Davis LE (2014). Combined autophagy and HDAC inhibition: a phase I safety, tolerability, pharmacokinetic, and pharmacodynamic analysis of hydroxychloroquine in combination with the HDAC inhibitor vorinostat in patients with advanced solid tumors. Autophagy.

[13] Rangwala R, Chang YC, Hu J, Algazy KM, Evans TL, Fecher LA (2014). Combined MTOR and autophagy inhibition: phase I trial of hydroxychloroquine and temsirolimus in patients with advanced solid tumors and melanoma. Autophagy.

[14] Rangwala R, Leone R, Chang YC, Fecher LA, Schuchter LM, Kramer A (2014). Phase I trial of hydroxychloroquine with dose-intense temozolomide in patients with advanced solid tumors and melanoma. Autophagy.

[15] Rosenfeld MR, Ye X, Supko JG, Desideri S, Grossman SA, Brem S (2014). A phase I/II trial of hydroxychloroquine in conjunction with radiation therapy and concurrent and adjuvant temozolomide in patients with newly diagnosed glioblastoma multiforme. Autophagy.

[16] Vogl DT, Stadtmauer EA, Tan KS, Heitjan DF, Davis LE, Pontiggia L (2014). Combined autophagy and proteasome inhibition: a phase 1 trial of hydroxychloroquine and bortezomib in patients with relapsed/refractory myeloma. Autophagy.

[17] McAfee Q, Zhang Z, Samanta A, Levi SM, Ma XH, Piao S (2012). Autophagy inhibitor Lys05 has single-agent antitumor activity and reproduces the phenotype of a genetic autophagy deficiency. Proc Natl Acad Sci USA.

[18] Ronan B, Flamand O, Vescovi L, Dureuil C, Durand L, Fassy F (2014). A highly potent and selective Vps34 inhibitor alters vesicle trafficking and autophagy. Nat Chem Biol.

[19] Bago R, Malik N, Munson MJ, Prescott AR, Davies P, Sommer E (2014). Characterization of VPS34-IN1, a selective inhibitor of Vps34, reveals that the phosphatidylinositol 3-phosphate-binding SGK3 protein kinase is a downstream target of class III phosphoinositide 3-kinase. Biochem J.

[20] Dowdle WE, Nyfeler B, Nagel J, Elling RA, Liu S, Triantafellow E (2014). Selective VPS34 inhibitor blocks autophagy and uncovers a role for NCOA4 in ferritin degradation and iron homeostasis in vivo. Nat Cell Biol.

[21] Koschmieder S, Gottgens B, Zhang P, Iwasaki-Arai J, Akashi K, Kutok JL (2005). Inducible chronic phase of myeloid leukemia with expansion of hematopoietic stem cells in a transgenic model of BCR-ABL leukemogenesis. Blood.

[22] Mizushima N, Yamamoto A, Matsui M, Yoshimori T, Ohsumi Y (2004). In vivo analysis of autophagy in response to nutrient starvation using transgenic mice expressing a fluorescent autophagosome marker. Mol Biol Cell.

[23] Komatsu M, Waguri S, Ueno T, Iwata J, Murata S, Tanida I (2005). Impairment of starvation-induced and constitutive autophagy in Atg7-deficient mice. J Cell Biol.

[24] Klionsky DJ, Abdelmohsen K, Abe A, Abedin MJ, Abeliovich H, Acevedo Arozena A (2016). Guidelines for the use and interpretation of assays for monitoring autophagy. Autophagy.

[25] Warr MR, Binnewies M, Flach J, Reynaud D, Garg T, Malhotra R (2013). FOXO3A directs a protective autophagy program in haematopoietic stem cells. Nature.

[26] Kimura S, Noda T, Yoshimori T (2007). Dissection of the autophagosome maturation process by a novel reporter protein, tandem fluorescent-tagged LC3. Autophagy.

[27] Kirkin V, Lamark T, Sou YS, Bjorkoy G, Nunn JL, Bruun JA (2009). A role for NBR1 in autophagosomal degradation of ubiquitinated substrates. Mol Cell.

[28] Mancias JD, Wang X, Gygi SP, Harper JW, Kimmelman AC (2014). Quantitative proteomics identifies NCOA4 as the cargo receptor mediating ferritinophagy. Nature.

[29] Holyoake T, Jiang X, Eaves C, Eaves A (1999). Isolation of a highly quiescent subpopulation of primitive leukemic cells in chronic myeloid leukemia. Blood.

[30] Brenner S, Ryser MF, Whiting-Theobald NL, Gentsch M, Linton GF, Malech HL (2007). The late dividing population of gamma-retroviral vector transduced human mobilized peripheral blood progenitor cells contributes most to gene-marked cell engraftment in nonobese diabetic/severe combined immunodeficient mice. Stem Cells.

[31] Weisberg E, Manley PW, Breitenstein W, Bruggen J, Cowan-Jacob SW, Ray A (2005). Characterization of AMN107, a selective inhibitor of native and mutant Bcr-Abl. Cancer Cell.

[32] Graham SM, Jorgensen HG, Allan E, Pearson C, Alcorn MJ, Richmond L (2002). Primitive, quiescent, Philadelphia-positive stem cells from patients with chronic myeloid leukemia are insensitive to STI571 in vitro. Blood.

[33] Zhang B, Ho YW, Huang Q, Maeda T, Lin A, Lee SU (2012). Altered microenvironmental regulation of leukemic and normal stem cells in chronic myelogenous leukemia. Cancer Cell.

[34] Ho TT, Warr MR, Adelman ER, Lansinger OM, Flach J, Verovskaya EV (2017). Autophagy maintains the metabolism and function of young and old stem cells. Nature.

[35] Liu F, Lee JY, Wei H, Tanabe O, Engel JD, Morrison SJ (2010). FIP200 is required for the cell-autonomous maintenance of fetal hematopoietic stem cells. Blood.

[36] Mortensen M, Soilleux EJ, Djordjevic G, Tripp R, Lutteropp M, Sadighi-Akha E (2011). The autophagy protein Atg7 is essential for hematopoietic stem cell maintenance. J Exp Med.

[37] Ma XH, Piao SF, Dey S, McAfee Q, Karakousis G, Villanueva J (2014). Targeting ER stress-induced autophagy overcomes BRAF inhibitor resistance in melanoma. J Clin Invest.

[38] Schemionek M, Elling C, Steidl U, Baumer N, Hamilton A, Spieker T (2010). BCR-ABL enhances differentiation of long-term repopulating hematopoietic stem cells. Blood.

[39] Rosenfeldt MT, O’Prey J, Morton JP, Nixon C, MacKay G, Mrowinska A (2013). p53 status determines the role of autophagy in pancreatic tumour development. Nature.

[40] Guo JY, Karsli-Uzunbas G, Mathew R, Aisner SC, Kamphorst JJ, Strohecker AM (2013). Autophagy suppresses progression of K-ras-induced lung tumors to oncocytomas and maintains lipid homeostasis. Genes Dev.

[41] Tothova Z, Kollipara R, Huntly BJ, Lee BH, Castrillon DH, Cullen DE (2007). FoxOs are critical mediators of hematopoietic stem cell resistance to physiologic oxidative stress. Cell.

[42] Zhang B, Li L, Ho Y, Li M, Marcucci G, Tong W (2016). Heterogeneity of leukemia-initiating capacity of chronic myelogenous leukemia stem cells. J Clin Invest.

[43] Rousselot P, Huguet F, Rea D, Legros L, Cayuela JM, Maarek O (2007). Imatinib mesylate discontinuation in patients with chronic myelogenous leukemia in complete molecular remission for more than 2 years. Blood.

