# Supplementary material for: Targeting quiescent leukemic stem cells using second generation autophagy inhibitors
Source: Leukemia. 2018 Sep 5;33(4):981–94. doi: 10.1038/s41375-018-0252-4 (PMC6292500; doi:10.1038/s41375-018-0252-4)
Supplement: Supplementary file 1 — Supplementary Figures + Figure Legends [file 41375_2018_252_MOESM1_ESM.pdf]

Supplementary Fig. S1

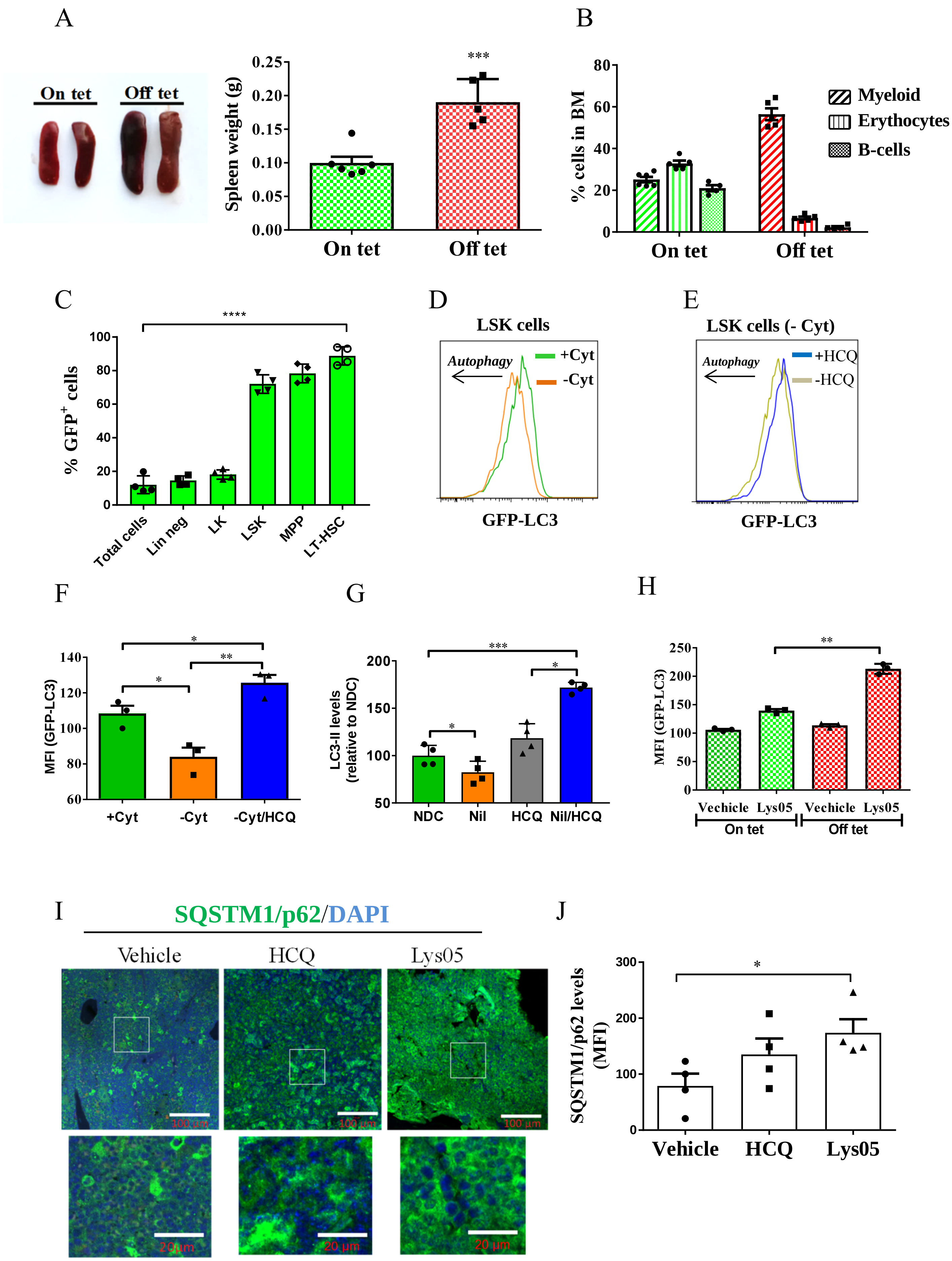

Fig. S1. Autophagy levels in whole BM following *in vivo* treatment with HCQ or Lys05. **(A)** Photograph and bar chart showing the change in spleen size and weight respectively after leukemia induction. Tetracycline was removed from *Scl-tTa-BCR-ABL* mice for 12 days (Off tet, n=5) while control (non-leukaemic) mice were kept on tetracycline (On tet, n=6). **(B)** Percentage of Gr1$^+$ Mac1$^+$ (myeloid), Ter119$^+$ (erythrocytes) and CD19$^+$ (B-cells) cells in BM of leukaemic mice (Off tet) and control mice (On tet). **(C)** Percentage of GFP$^+$ cells in BM populations of non-leukemic mice. **(D)** Representative histogram showing GFP-LC3 levels in LSK cells following 6h withdrawal of cytokines from the culture media. **(E)** Representative histogram showing GFP-LC3 levels in LSK cells in the presence or absence of HCQ (20µM) following cytokine withdrawal. **(F)** Quantitative analysis of GFP-LC3 levels following cytokine withdrawal in the presence or absence of HCQ. Results are shown relative to LSK cells cultured in complete media (+ cytokines). Error bars represent +/– SEM. **(G)** Quantitative analysis of LC3-II in LSK cells following 6h *in vitro* treatment with nilotinib (2µM), HCQ (20µM) and combination. **(H)** Bar plot representing GFP-LC3 levels in LSK cells, isolated from non-leukemic and leukemic mice, in the presence or absence of 6h Lys05 (10µM) treatment**.** **(I)** Representative images showing SQSTM1/p62 levels measured in whole BM sections of mice treated for 2 days with vehicle (PBS; n=4), HCQ (n=4) or Lys05 (n=4). **(J)** Quantification of SQSTM1/p62 levels in BM sections shown by mean fluorescence intensity (MFI). Results are shown as relative to no-drug control (NDC). Error bars represent +/– SD (A, C, G, H) or SEM (B, D-F, J).

Supplementary Fig. S2

A

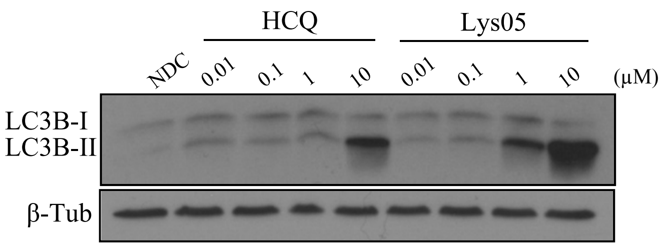

B

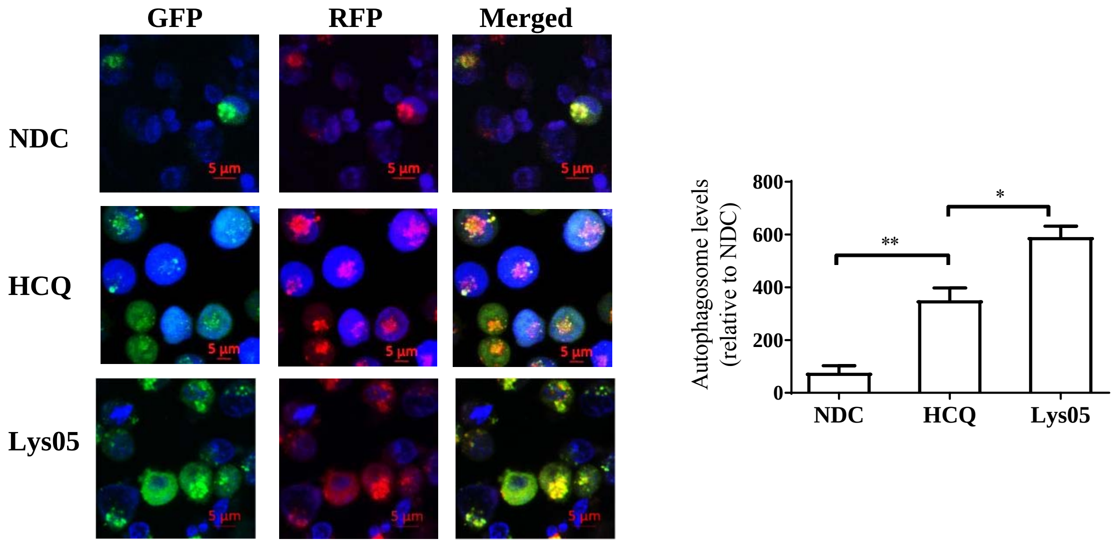

C

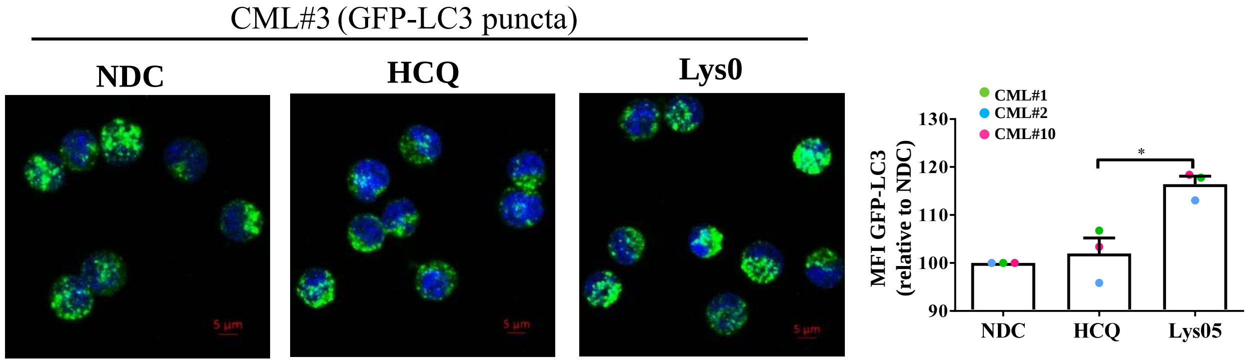

Fig. S2. Autophagy levels in leukaemic cells including CD34$^+$ primary cells following *in vitro* treatment with HCQ or Lys05. (A) Levels of LC3-I and LC3-II in KCL22 cells after 4h treatment with vehicle (NDC), HCQ or Lys05 at increasing concentrations from 0.01 to 10µM. β-tubulin was used as loading control. **(B)** Representative confocal fluorescent images of KCL22 cells expressing mRFP-GFP-LC3 following 4h treatment with HCQ (5µM), Lys05 (5µM) or vehicle (NDC). Quantification of autophagosome levels were calculated using the co-localization coefficient between red (RFP) and green (GFP). 3 independent experiments were performed. Error bars represent +/- SD. **(C)** Representative image of CD34$^+$ CML cells immunostained with anti-LC3 antibody after 72h treatment with vehicle (NDC), HCQ (3µM) or Lys05 (3µM). LC3 puncta were quantified by measuring the MFI of GFP. Error bars represent +/– SEM. n=3 individual patient samples.

Supplementary Fig. S3

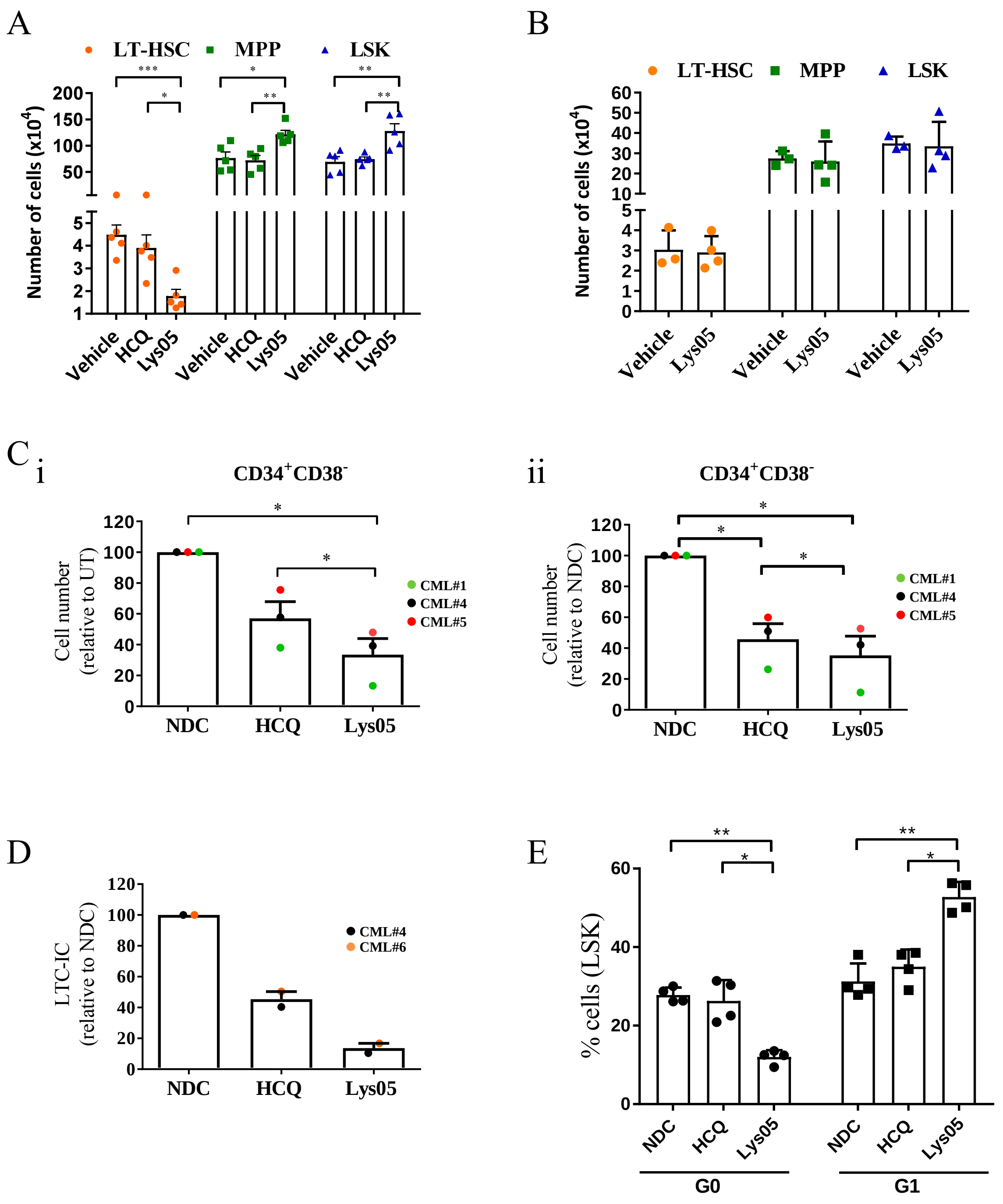

Fig. S3. *In vitro* effects of HCQ and Lys05 on LSCs. (A-B) Representative plots showing the absolute numbers of cells in LSK, MPP and LT-HSC populations in the BM of leukemic **(A**; n=5 per treatment**)** and non-leukemic **(B**; n=4 per treatment**)** mice following 2 days of *in vivo* treatment with vehicle (PBS), HCQ or Lys05. **(C)** Cell number of $CD34^{+}CD38^{-}$ cells following 3 **(i)** and 6 **(ii)** days treatment with vehicle (NDC), HCQ (5µM) or Lys05 (5µM) (n=3 patient samples). **(D)** Number of colonies obtained by LTC-IC assays following *in vitro* treatment of $CD34^{+}$ cells (n=2 patient samples) for 6 days with vehicle (NDC), HCQ (5µM) or Lys05 (5µM). **(E)** Percentage of cells in G0 and G1 cell cycle phases following 72h *in vitro* treatment of pooled LSK cells isolated from leukemic mice (n=5) with vehicle (NDC), HCQ (3µM) and Lys05 (3µM).

Supplementary Fig. S4

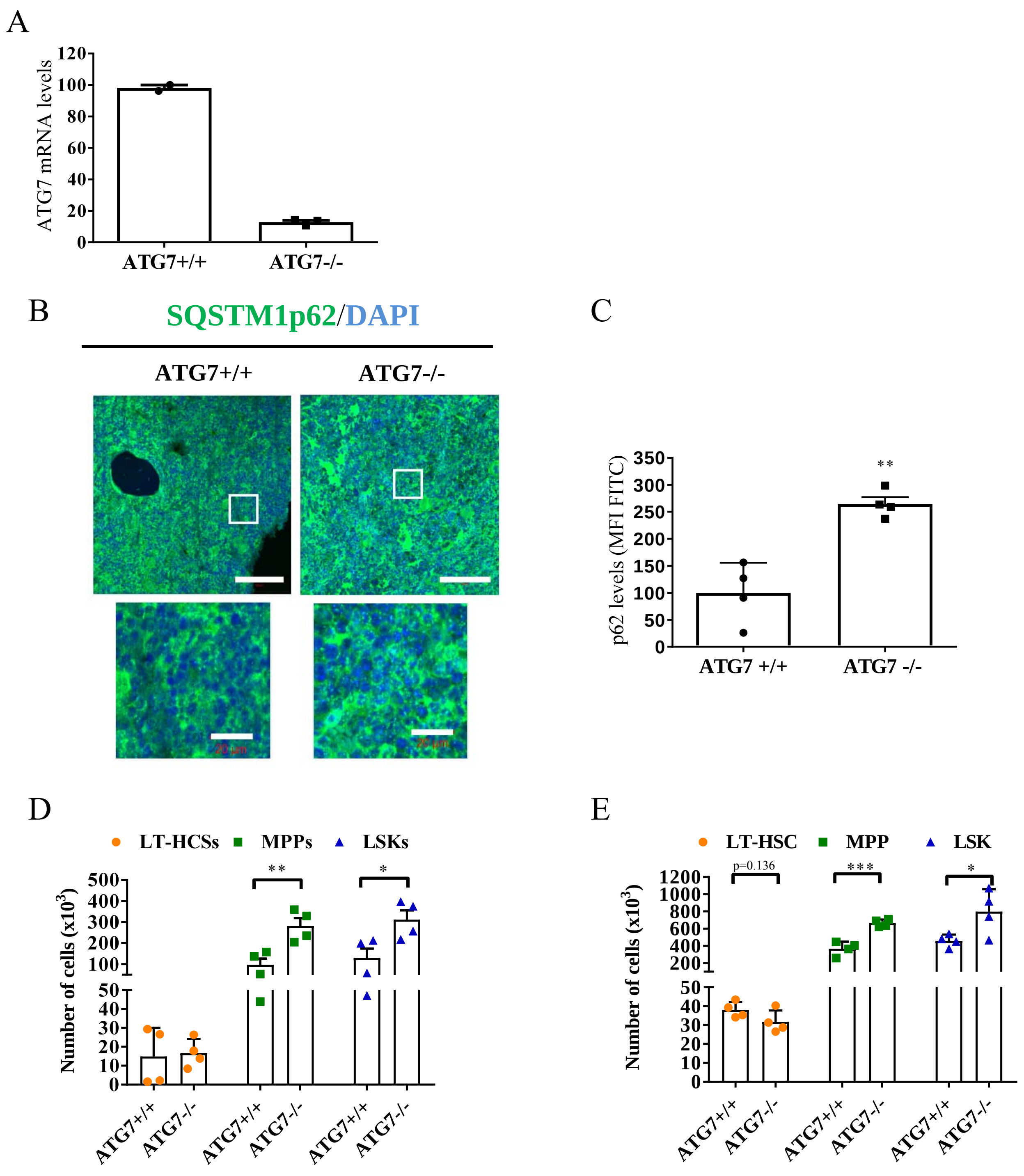

Fig. S4. Expansion of MPPs and LSK cells in autophagy deficient *Scl-Tta-BCR-ABL* mice. **(A)** Relative ATG7 mRNA levels in LK cells from leukaemic mice with *Atg7* ($ATG7^{+/+}$) or without *Atg7* ($ATG7^{-/-}$) mice. **(B)** Representative images of SQSTM1/p62 expression on whole BM sections of $ATG7^{+/+}$ and $ATG7^{-/-}$ mice. **(C)** Quantification of SQSTM1/p62 levels on whole BM of $ATG7^{+/+}$ (n=4) and $ATG7^{-/-}$ mice (n=4). Results are represented relative to the MFI values obtained from $ATG7^{-/-}$ mice **(D-E).** Number of LT-HSC, MPP and LSK cells in the BM of leukemic (**D**) and non-leukaemic (**E**), $ATG7^{+/+}$ (n=4) and $ATG7^{-/-}$ mice (n=4). Error bars represent +/– SEM.

Supplementary Fig. S5

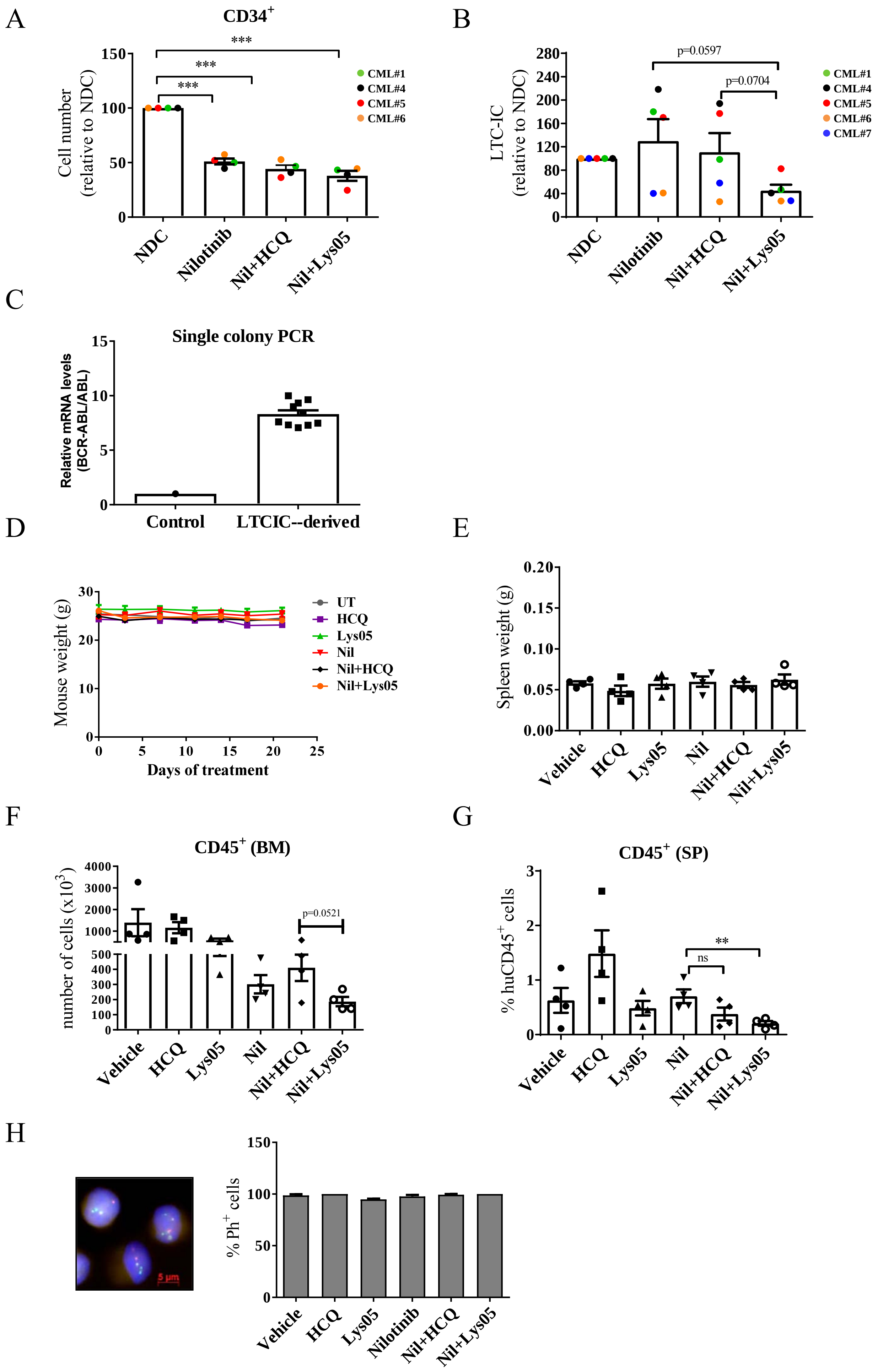

Fig. S5. Effects of combined inhibition of autophagy and BCR-ABL on LSCs *in vitro* and *in vivo.* **(A)** Number of $CD34^+$ cells following 6-day *in vitro* treatment with vehicle (NDC), nilotinib as a single agent (2µM), or combination of nilotinib with HCQ (5µM) or Lys05 (5µM), n=4 individual patient samples. **(B)** Number of colonies obtained from LTC-IC assays performed with $CD34^+$ cells previously treated for 3 days with vehicle (NDC), nilotinib as a single agent (2µM), or combination of nilotinib with HCQ (3µM) or Lys05 (3µM). n=5 patient samples. Results are represented relative to NDC. Error bars represent +/– SEM. **(C)** Representative real-time quantitative PCR for BCR-ABL expression from individual colonies obtained from $CD34^+$ CML LTC-IC**.** **(D)** Weight of NSG mice at different time points during the *in vivo* treatment. **(E)** Spleen weight of NSG mice at end-point showing no significant changes between the experimental arms. Error bars represent +/– SEM. **(F and G)** Absolute number of $CD45^+$ cells extracted from the BM (F) or spleen (G) of NSG mice following *in vivo* treatment. **(H)** Representative image and quantification of human $CD45^+$ cells expressing BCR-ABL detected by D-FISH (Vehicle, n=2; HCQ, n=2; Lys05, n=3, nilotinib, n=3; nilotonib+HCQ, n=2; nilotinib+Lys05, n=2). Results are represented by percentage of Ph^+ in the BM of NSG mice.

Supplementary Fig. S6

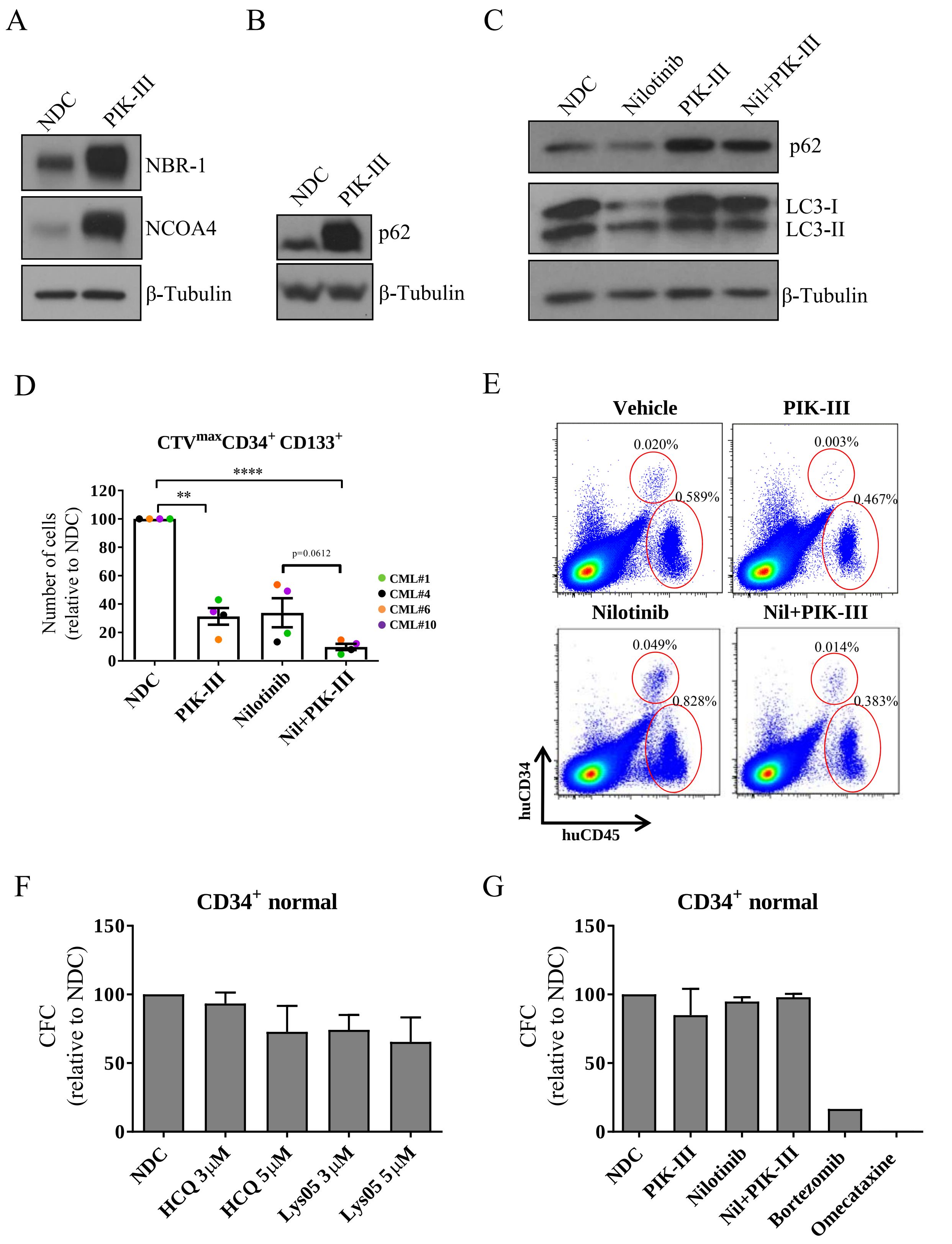

Fig. S6. Effects of the specific autophagy inhibitor PIK-III on LSCs. (A and B) Blots showing the levels of the autophagy substrates NBR1, NCOA4 (A) and SQSTM1/p62 (B) in CD34$^+$ cells following 72h treatment with vehicle (NDC) or PIK-III (5µM). **(C)** Western blot showing SQSTM1/p62 and LC3I/II levels in CD34$^+$ cells following 24h treatment with Nilotinib (2µM), PIK-III (5µM) or both drugs combined. β-tubulin was used as loading control. Results are representative of 3 independent experiments performed with 3 separate patient samples. Results are shown relative to NDC. **(D)** Number of CTV^{max}CD34$^+$CD133$^+$ cells following *in vitro* treatment for 3 days with vehicle (NDC), PIK-III (5µM), Nilotinib (2µM) or combination of PIK-III with nilotinib. **(E)** Representative plots showing CD45$^+$CD34$^+$ populations from each arm of the experiment (Vehicle, n=4; PIK-III, n=5; nilotinib, n=5; nilotinib + PIK-III, n=5). **(F)** Number of colonies from CFC assays performed on non-CML CD34$^+$ cells (n=3) following treatment with HCQ (3 and 5µM), Lys05 (3 and 5µM) or vehicle (PBS). **(G)** Colony number obtained by CFC assays from non-CML CD34$^+$ samples (n=3) following treatment with PIK-III (5µM), nilotinib (2µM), the combination of PIK-III and nilotinib, bortezomib (20nM) and omacetaxine (10nM). Results are represented as relative to NDC. Error bars represent +/– SEM.

Supplementary Fig. S7

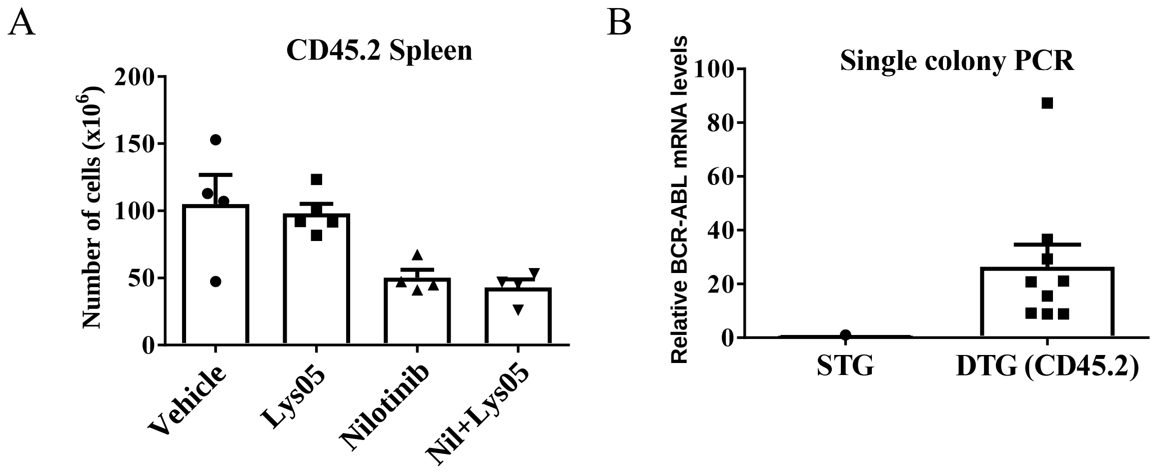

Fig. S7. Assessment of disease burden in primary transplanted mice following *in vivo* treatment. (A) Absolute number of CD45.2$^+$ cells in the spleens of leukaemic primary transplanted mice after treatment **(B)** Real-time quantitative PCR for BCR-ABL expression in single colonies derived from leukemic LSK-CD45.2 cells, relative to non-leukemic control. Error bars represent +/– SEM.
