# Supplementary material for: Targeting quiescent leukemic stem cells using second generation autophagy inhibitors
Source: Leukemia. 2018 Sep 5;33(4):981–94. doi: 10.1038/s41375-018-0252-4 (PMC6292500; doi:10.1038/s41375-018-0252-4)
Supplement: Supplementary file 2 — Supplementary Methods [file 41375_2018_252_MOESM2_ESM.docx]

**Supplemental Methods**

*In vivo studies*

For GFP-LC3 detection, tetracycline was removed for 10-15 days followed by HCQ or Lys05 treatment for 2 or 7 days. HCQ (32 mg/kg) and Lys05 (20 mg/kg) were injected daily once per day intraperitoneally (IP). Stock solutions were made in PBS (5mg/mL for HCQ and 2.5mg/mL for Lys05). For conditional deletion of *Atg7*, *Atg7^flox/flox^:Mx-Cre/BCR-ABL or* *Atg7^flox/flox^:Mx-Cre,* mice were injected four times intraperitoneally every 2 days with 250µL/mouse of 1mg/mL poly(I:C). Expression of BCR-ABL was simultaneously induced and *Scl-tTa–BCR-ABL* mice injected with PBS were used as controls. For experiments with non-leukemic mice *Atg7^flox/flox^:Mx-Cre^-^* mice were used as controls and also treated with poly(I:C).

For primary transplants BM cells were collected from femurs and hips from CD45.2 *Scl-tTa–BCR-ABL* mice and 2x10^6^ cells transplanted via tail vein into pre-irradiated CD45.1 WT recipients (2 x 4.25Gy, 3 hours apart) aged 8-12 weeks. Mice were kept on tetracycline for 3 weeks before disease induction. Leukemia was induced for 4 weeks followed by 3 weeks treatment with nilotinib (oral gavage, 75 mg/kg/day), Lys05 (IP, 32 mg/kg/day) or both drugs combined. Nilotinib was prepared in 10% NMP-90% PEG300 at 10mg/mL and Lys05 in PBS at 2.5mg/mL. The appropriate vehicles were administered when required according to experimental conditions.

For secondary transplants, a pool of BM cells from each experimental arm was used. 3x10^6^ cells per mouse were transplanted into CD45.1 WT secondary recipients, pre-irradiated as previously detailed. Disease progression was monitored weekly by Gr1-1(Ly-6G/Ly-6C) and Mac-1 (CD11b) (BioLegend) detection in blood. Mice with similar levels of myeloid cells were used for all experimental arms. At the end of the treatments, BM cells were collected by crushing legs and pelvic bones in PBS containing 2% FBS. Spleens were processed by mechanical dissociation and both BM cells and splenocytes were filtered using 40µm cell strainers (EASYstrainer, Greiner Bio-One).

For purification of LSK and LK cells, BM cells were enriched for c-Kit^+^ cells using c-Kit microbeads and midiMACS Separator kit with Separation LS Columns (all from Miltenyi Biotec). For LSK sorting, c-kit^+^ cells were incubated with Fc block (CD16/CD32 Fcγ III/II Receptor, BD Bioscience) followed by staining with a linage antibody cocktail (CD4, CD5, CD8a, Mac-1, B220, Ter119 and Gr1; all biotinylated and from BD Bioscience), Sca-1-PE-Cy7 and c-kit APC-Cy7 antibodies (Biolegend). Cells were sorted using a FACSAria Fusion Cell sorter (BD Biosciences) following staining with Streptavidin-PB (Life Technologies).

For LT-HSC staining, whole BM and/or spleen cells were stained with the linage cocktail containing the additional antibodies: CD45.2-PerCP-Cy5.5, CD45.1-FITC, Sca-1-PE-Cy7, c-kit APC-Cy7, CD45-PE and CD150-APC (Biolegend). For assessment of engraftment and disease levels, whole BM cells were incubated with Fc block followed by staining with anti-CD45.2-PB, anti-CD45.1-FITC, anti-Gr1-APC, anti-Mac-1-PE, anti-Ter119-APC-Cy7 and anti-CD19-PE-Cy7 (all Biolegend).

For the assessment of engraftment followed by *in vivo* treatment, 1×10^6^ CD34^+^ CML cells were transplanted via tail vein into sub-lethally irradiated (2.25 Gy) female NSG mice (NOD.Cg-*Prkdc*^scid^ *Il2rγ*^tm1Wjl^/SzJ; The Jackson Laboratory) aged 8 weeks. Twelve weeks following transplant, mice were treated daily once per day with nilotinib (oral gavage, 50mg/kg), Lys05 (IP, 20mg/kg) or combination for 3 weeks. For xenografts following *in vitro* treatments, CD34^+^ CML cells were cultured in the absence of drug (vehicle) or with addition of nilotinib (2µM), PIK-III (5µM) or both for 48h. Cells were then harvested, washed and transplanted into sub-lethally irradiated (2.25 Gy) female NSG mice aged 8 weeks. At the endpoint, BM cells were stained with anti-human CD45-FITC (BD Biosciences), anti-human CD34-APC (BD Biosciences), anti-human CD38-PerCP (Biolegend), anti-human CD133-PE (Miltenyi Biotec) antibodies for flow cytometry analysis. All flow cytometry experiments were carried out using the BD FACSVerse^TM^ Flow Cytometer (BD Bioscience). Data analysis was performed using FlowJo 7.6.5 software.

*Cell culture*

Chronic phase CD34^+^ CML and CD34^+^ non-CML cells were cultured in serum-free medium (SFM) comprising Iscove’s modified Dulbecco’s medium (IMDM) supplemented with 20% BIT (bovine serum albumin/ insulin/transferrin; StemCell Technologies), 1% L-glutamine (Invitrogen), 1% (vol/vol) streptomycin/penicillin (Invitrogen), 40 µg/ml low-density lipoprotein and 0.1 mM 2-mercaptoethanol (Invitrogen). This medium was further supplemented with a physiological growth factor (PGF) cocktail; 0.2 ng/mL SCF/GM-CSF/MIP-α, 1.0 ng/mL G-CSF/IL6 (PeproTech EC Ltd) and 0.05 ng/mL LIF, (StemCell Technologies). Murine LSK cells were cultured in SFM consisting of IMDM, 20% BIT, 1% L-glutamine, 1% (vol/vol) streptomycin/penicillin, 0.1 mM 2-mercaptoethanol, 50ng/mL SCF, 10 ng/mL IL-3, 25 ng/mL IL-6 (PeproTech EC Ltd) and 0.05 ng/mL LIF. The CML cell lines K562 and KCL22 (DSMZ) were cultured in RPMI 1640 medium (Life Technologies) supplemented with 1% (vol/vol) penicillin/streptomycin, 1% L-glutamine and 10% (vol/vol) foetal calf serum (Life technologies) and routinely tested to ensure they were free of mycoplasma.

*Cell cycle analysis*

Permeabilization was performed overnight with 0.2% triton in PBS. FITC Mouse Anti-Ki-67 Set (BD Bioscience) was used for staining according to manufacturer’s instructions. 7-AAD (BD Bioscience) was used as nucleic acid dye. Cell analysis was performed with FACSVerse^TM^ Flow Cytometer (BD Bioscience) and data was analysed using FlowJo 7.6.5 software.

*Dual-fusion interphase fluorescence in situ hybridization (D-FISH)*

Human CD45*^+^* cells from NSG xenografts were scored for the presence of the Ph chromosome. FISH was performed with the LS1 BCR–ABL Dual Color, Dual Fusion translocation probe according to the manufacturer’s instructions (Abbott Diagnostics). Two hundred cells were scored from BM from each xenograft.

*In vitro treatments*

10mM stock solutions of HCQ sulfate (Sigma) and Lys05 were prepared in PBS and stored in aliquots at -20°C. PIK-III was prepared in DMSO and store in aliquots of 5mM at -20°C. Nilotinib was either provided by Novartis Pharma (Basel, Switzerland) or purchased from LC Laboratories (N-8207). Stock solutions of 10mM and 2mM nilotinib were prepared in DMSO and stored in aliquots at -20°C. To compare the potency between HCQ and Lys05, equimolar concentrations were used in each experiment. All concentrations used for *in vitro* treatments are indicated in the figure legends.

*CFC and LTC-IC assays*

For both assays, primary CD34^+^ CML cells were cultured for 3 or 6 days in the presence or absence of the indicated drugs. For CFC assays, 5×10^3^ primary cells from each condition were added into 3mL of methylcellulose-based medium (Methocult H4034 Optimum, StemCell Technologies). 1.5 mL was transferred to a 35 mm tissue culture dish in duplicate and the number of colonies was counted after 10-14 days. For LTC-IC, cells were cultured on irradiated stromal feeder layers (M2-10B4 and S1/S1) and kept in culture with MyeloCult (StemCell Technologies) for 5 weeks. Cells were then transferred to CFC assays in duplicate and viable colonies were counted after 10-14 days.

*Immunofluorescence of bone sections*

Murine femurs were fixed in 10% neutral buffered formalin (NBF), decalcified and subsequently embedded in paraffin. For staining, 3µm femur sections were dewaxed in xylene and rehydrated in an ethanol to water graded series. Antigen retrieval was performed overnight at pH 9 (Retrieval solution TRS9, Dako, 60°C, O/N) before blocking in PBS/3% BSA/10%FBS (GE Healthcare). Tissue-sections were then incubated with anti-SQSTM1/p62 (Sigma), followed by incubation with Alexa fluor 488-conjugated secondary antibodies (Invitrogen). Stained tissue sections were mounted using Prolong Gold Antifade reagent with DAPI (Invitrogen). 10 images (20x) were taken per mouse and at least three mice were analysed per experimental condition. Images were visualized using a Zeiss LSM 780 confocal microscope and analysis was performed with ZEN2.1/ZEN 2 software.

*LC3 puncta, RFP-GFP-LC3 and LC3-II detection*

For LC3 puncta detection, CD34^+^ CML cells were plated on 0.01% poly-l-lysine (Sigma) pre-coated multispot microscope slides and, after 90min, fixed in PBS/3.7% formaldehyde for 20min at room temperature. Cells were permeabilised in PBS/0.5% Triton-X-100 for 15min at room temperature. Slides were then washed with PBS and incubated with blocking solution (PBS/5% BSA) for 1h at room temperature followed by overnight incubation with LC3B antibody (Cell signalling) at 4°C. Slides were then washed with PBS and incubated with either Alexa Fluor 488 donkey anti-rabbit (Invitrogen) or the isotype control (Rabbit IgG, abcam) for 1h at room temperature. Mounting media with DAPI (Vectashield) was used for nuclei staining and slides were analysed using Zeiss LSM 780 confocal microscope. Analysis of the data was performed using the Imaging Software ZEN 2.1. For each of the patient samples, 5 images (63x) were taken from each condition and 10 cells were analysed per individual image. Each experimental condition was performed in duplicate (technical replicate). K562 and KCL22 cells expressing mRFP-GFP-LC3 were used to measure autophagy flux by confocal microscopy. Cells were plated on poly-l-lysine pre-coated multispot microscope slides and after 90min were washed with PBS and fixed with PBS/3.7% formaldehyde for 15min at room temperature. Two more washes were performed before visualizing the images in a Zeiss LSM 780 confocal microscope. For each of the samples 10 images were taken from each condition and 10 cells were analysed per individual image. All images were analysed with ZEN2.1/ZEN 2 software. For measuring autophagy flux in LSK cells by LC3-II detection *FlowCellect™ Autophagy LC3 Antibody-based Assay Kit* (MerckMillipore) was used according to manufacturer’s instructions. Cell analysis was performed with FACSVerse^TM^ Flow Cytometer (BD Bioscience) and data analysed using FlowJo 7.6.5 software.

*Western blot analysis*

Whole-cell extracts were prepared by lysing cells in RIPA buffer (Thermo Fisher Scientific) 1% SDS containing protease and phosphatase inhibitors (Roche). Extracts were separated by SDS-PAGE, transferred to PVDF membranes and incubated with the following antibodies: LC3B (Cell Signaling), SQSTM1 (BD Biosciences), NBR1 (Abnova) and ARA-70 (SCBT). Primary antibodies were detected by enhanced chemiluminescence (GE Healthcare/Amersham) using a horseradish peroxidase−linked secondary antibody (Cell Signaling Technology).

*RNA extraction and quantitative PCR*

Total RNA was extracted and purified using PicoPure RNA Isolation Kit (Applied Biosystems, 12204) and retrotranscribed to cDNA using a high-capacity cDNA Archive Kit (Applied Biosystems). *Atg7* expression in LK cells and whole bone marrow of non-leukemic mice was measured by real-time PCR using Taqman Universal PCR Master Mix with specific primers for *Atg7* (Applied Biosystems, Mm00512209) and GAPDH (Applied Biosystems, Mm 99999915). For BCR-ABL detection in single colonies, PCR reaction was performed using the following probe and set of primers: ENP541* (CCCTTCAGCGGCCAGTAGCATCTGA); ENF501* (TCCGCTGACCATCAAYAAGGA) and ENR561* (CACTCAGACCCTGAGGCTCAA). The PCR reactions were performed in the CFX96 Touch Real-Time PCR Detection System (BIO-RAD). *ENP=European network TaqMan probe, ENF=European network forward primer, ENR=European network reverse primer.

*Statistics*

For the *in vivo* work, the investigators were blinded to the experimental conditions when assessing the outcomes. All mice were cared for equally in an unbiased fashion by animal technicians and investigators and no animal was excluded from the analysis*. P* values were calculated by two-tailed paired or unpaired *t*-tests for *in vitro* or *in vivo* experiments respectively, using the software Prism 5.0 (GraphPad Inc.). Biological variation for both human samples and animals is indicated by the error bars and was calculated for each group of data as means ± SD or SEM. Significance is indicated as follows: **p*<0.05, ***p*<0.01 and ****p*<0.001.

*Study approval*

Animal work was carried out with ethical approval from the University of Glasgow Animal Welfare and Ethical Review Board (AWERB) under Home Office License PPL 60/4492. Informed consent for all human samples was obtained in accordance with the Declaration of Helsinki and approval of the National Health Service (NHS) Greater Glasgow Institutional Review Board and Clyde Biorepository. Ethical approval for this work has already been granted by the West of Scotland Research Ethics Service (REC reference: 15/WS/0077).
